# AP-3 complex sorts preferential cargo to govern dense core vesicle function in neuroendocrine cells

**DOI:** 10.1016/j.jbc.2026.113396

**Published:** 2026-08-05

**Authors:** Shashank Saxena, Chandramouli Mukherjee, Vinayak Ghosh, Badal Singh Chauhan, Bhavani Shankar Sahu

**Affiliations:** 1 National Brain Research Centre, Manesar, Gurgaon, Haryana, India; 2 Department of Biological Sciences, Tata Institute of Fundamental Research, Mumbai, Maharashtra, India

## Abstract

This study reveals the multifaceted role of the adaptor protein (AP-3) complex in the function of dense-core vesicles (DCVs). Despite numerous studies, an existing lack of knowledge concerning the role of AP-3 in DCV function prompted us to delve deeper. Biochemical analysis revealed compromised DCV exocytosis in AP-3-depleted PC12 cells. AP-3 depletion altered the size and positioning of DCVs. Golgi defects and RUSH (retention using selective hook) substantiated the role of AP-3 in trans-Golgi DCV sorting. Proteomics analysis of cellular fractions revealed that AP-3 depleted cells showed the loss of specific DCV proteins, accompanied by mislocalization and rerouting to lysosomes. Bioinformatics and proximity ligation assays revealed interactions of mislocalized proteins with the AP-3 subunit. These findings corroborated functional defects in granule maturation, release modes, zinc, and neurotransmitter mobilization. Our study demonstrates multifaceted roles of the AP-3 complex in governing DCV function, thereby enabling us to understand neurotransmitter and neuropeptide release.

Adaptor protein (AP) complexes are heterotetrameric coat protein complexes that dictate the loading of membrane lipids, membrane proteins, and components for vesicular formation and fusion at the Golgi, endosomes, or plasma membrane (1, 2, 3). APs are paralogous groups of proteins composed of distinct subunits, constituting five different APs (AP1–AP5) (4, 5, 6, 7). AP-3 consists of two larger subunits, identified as β and δ, and two smaller subunits, identified as μ and σ, where the δ subunit is unique to the AP-3 complex (8). The β subunit of AP-3 has ubiquitously expressed (A) and neuron-specific (B) isoforms, where the neuron-specific form is crucial for the biogenesis and release of Dense core vesicles (DCVs) and synaptic vesicles (9, 10). APs that interact with DCV cargo proteins involve tyrosine-based sorting motifs (YXXΦ; where X can be a polar residue and Φ is a bulky hydrophobic residue) (11) as well as dileucine motifs ([DE]xxxL[LI]; consisting of acidic residues, where X can be any amino acid) at the cytoplasmic tail (4). The AP-3 complex is recruited to the trans-Golgi network (TGN) and endosomal compartments (12) with the help of ADP-ribosylation factor 1 (ARF1) (13), which participates in the transport of cargo towards the lysosome (14) and lysosome-related organelles such as melanosomes and platelets (15, 16). Work related to AP-3 has led to the identification of two naturally occurring mutant mice lacking either the AP-3 δ subunit (17) or the β subunit (18). Mocha mice have a null mutation in AP-3 δ and, therefore, lack functional AP-3 (17). While Pearl mice have mutations in the AP-3’s ubiquitously expressing A isoform of the β subunit, the brain-specific B isoform is unaltered (18). AP-3 deficiency in mocha mice impairs stimulus-coupled DCV release in adrenal chromaffin cells and increases their quantal size, reversed upon AP-3 expression (19, 20). While previous reports indicate defective biogenesis and impaired exocytosis of DCVs in the absence of AP-3 (19, 20, 21), the precise mechanisms through which AP-3 influences specific cargoes and regulates other DCV functions are intriguing and worthy of further investigation.

We have used a multidisciplinary approach to address these understudied aspects of the AP-3 complex in regulating DCV function. First, we disrupted AP-3 function by targeting its core δ subunit using multiple shRNAs (22). This lentiviral knockdown approach is advantageous over other loss-of-function approaches and provides a more controlled and efficient system for studying gene function compared to traditional rescue experiments (23, 24). Functional analyses of DCVs, including biochemical assays, revealed compromised DCV exocytosis in AP-3-depleted cells. Ultrastructural transmission electron microscopy (TEM) and morphometric analysis further revealed an increased proportion of immature DCVs positioned farther away from the plasma membrane. This finding was consistent with increased colocalization of DCVs with syntaxin 6, a marker of immature DCVs that are typically trafficked back to the TGN after maturation (25). The role of AP-3 in trans-Golgi DCV sorting was further elucidated using retention using selective hook (RUSH) and brefeldin A (BFA). To broaden our understanding, we conducted comparative quantitative organellar proteomics of vesicle-enriched fractions (VEF), as previously described (22). Our analysis demonstrated the depletion of key membrane proteins associated with DCVs, including known markers (SYT1, VMAT1, ZnT3) (26, 27, 28) and putative novel candidates (DLK1, SCN3β). Confocal microscopy revealed the mislocalization of these proteins to lysosomes rather than DCVs, suggesting that AP-3 deficiency leads to missorting and rerouting of selective DCV cargo to lysosomes. Bioinformatics analysis identified binding motifs in these candidate AP-3 subunit proteins, further supporting their dependence on AP-3 for proper sorting.

Functionally, AP-3 depletion compromises DCV compartments, as evidenced by reduced neurotransmitter and zinc mobilization—hallmarks of mature DCVs (29, 30). These functional defects were attributed to the mislocalization of VMAT1 and ZnT3. Additionally, we observed a significant reduction in fusion pore dilation, likely due to missorting of SYT1, a calcium sensor associated with the fusion pore complex. In summary, our study demonstrated that the AP-3 complex plays a critical, multifaceted role in sorting proteins into the DCV compartment. Future studies should explore its interactions with other adaptor protein complexes, such as AP-1, which has been implicated in DCV biogenesis (31, 32). Moreover, the specific cargo sorting pathways of the AP-3 complex—from the trans-Golgi network or recycling endosomes to DCVs—require further investigation, which we plan to address in future work.

## Results

### Generation and validation of stable and inducible AP-3 knockdown PC12 cells

**Figure 1:**
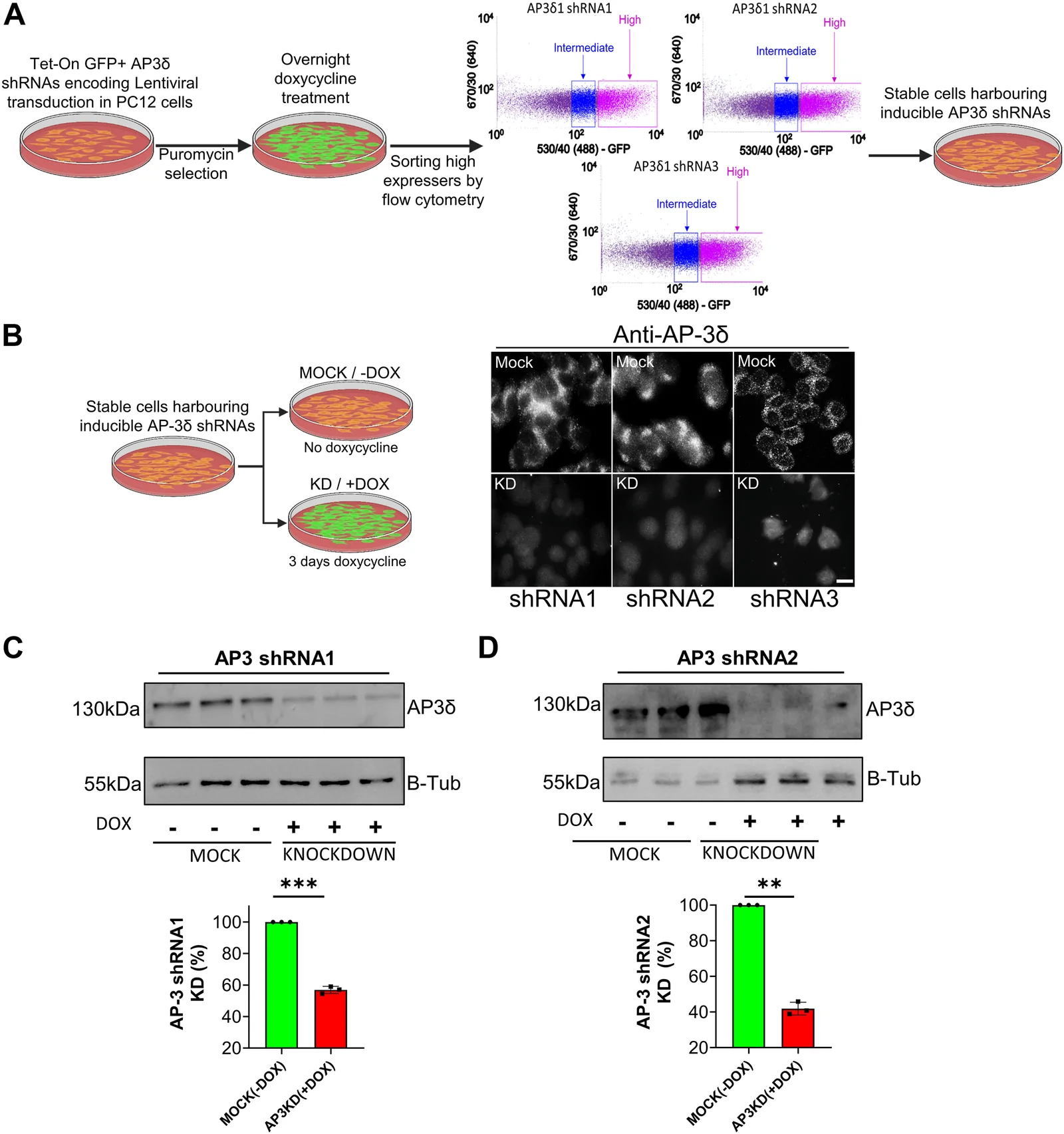
**Generation and validation of stable and inducible AP-3 knockdown PC12 cells.***A*, schematic of the strategy to generate inducible AP-3 knockdown cells. PC12 cells were transduced with SMARTvector lentiviral shRNAs (sh1, sh2, and sh3) co-expressing GFP targeting the δ1 subunit of the AP-3 complex. Cells were treated with doxycycline for 24 h to induce shRNA expression, followed by withdrawal of doxycycline and enrichment of high GFP-expressing cells by flow cytometric sorting. *B*, selection was carried out with puromycin to obtain a stable population of cells expressing inducible AP-3 shRNAs. To validate AP-3 Knockdown, cells were mock-treated or treated with doxycycline for 3 days and subjected to immunofluorescence. *C-D*, immunoblots and Quantification for AP-3shRNA1 and AP-3shRNA2 cells indicating a significant depletion upon knockdown (n = 3, two-tailed unpaired *t* test with Welch’s correction, ∗∗∗, *p* = 0.0009, ∗∗, *p* = 0.0013 respectively). Scale bar: 10 μm.

AP-3 is a heterotetrameric complex in which targeting the δ subunit allows maximum disruption of the AP-3 complex, as the δ subunit trunk forms the core of the AP-3 complex (33). We developed a doxycycline-inducible Tet-on knockdown system in PC12 cell lines targeting the δ subunit of AP-3 to enable temporal loss of the AP-3 complex. We designed three unique guides targeting different regions of the AP-3 δ subunit, and a mixed population of cells with high expression was FACS-sorted and stably selected for each guide, respectively (Fig. 1*A*), and established as knockdown cells for all subsequent experiments (see Materials and methods). All three knockdown (KD) cell lines showed significant depletion of AP-3 expression upon 3-days doxycycline induction compared with mock-treated cells, as shown by immunofluorescence images and immunoblots (Figs. 1, *B*–*D* and S1*A*).

### Stimulus-coupled secretion of DCV cargo revealed the regulated secretion of DCVs to be compromised in AP-3-depleted cells

**Figure 2:**
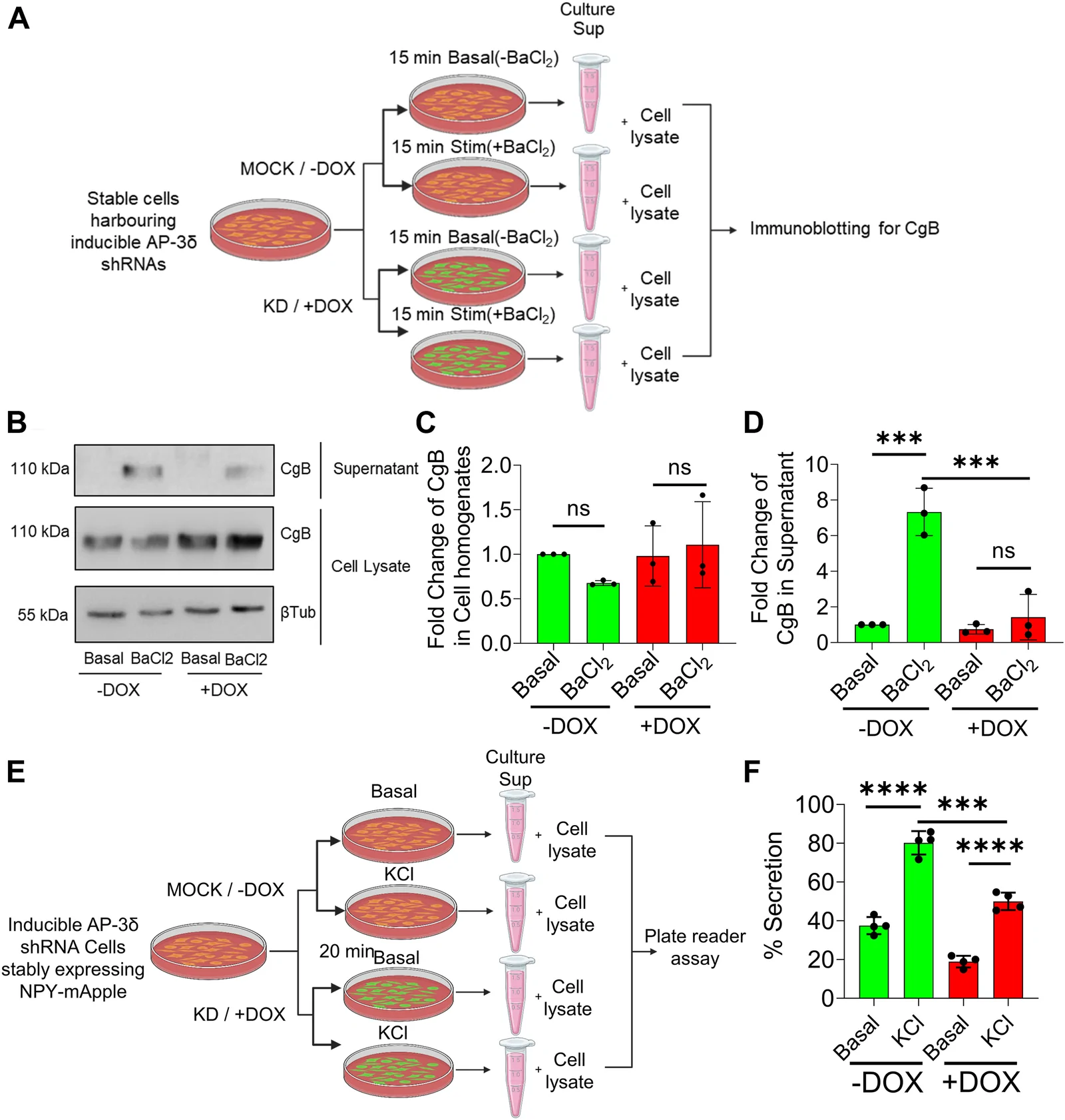
**Stimulus-coupled secretion of DCV cargo revealed the regulated secretion of DCVs to be compromised in AP-3 depleted cells.***A*, schematic of the secretion assay. Mock treated (−DOX) and AP-3 depleted (+DOX; 3 days doxycycline) PC12 cells were analyzed for chromogranin B (CgB) secretion. *B*, representative Western blot showing CgB levels in cell homogenate and culture supernatant under basal or BaCl_2_ stimulated conditions. *C*, quantification of CgB in cell homogenates revealed no significant differences between −DOX and +DOX cells in either basal or stimulated conditions (two-tailed unpaired *t* test). *D*, quantification of secreted CgB in culture supernatants demonstrated robust BaCl_2_-dependent release in −DOX cells, whereas +DOX cells failed to secrete CgB upon stimulation, resulting in a significant reduction compared with mock treated (−DOX) cells (N = 3; one-way ANOVA with Tukey’s *post hoc* test, ∗∗∗, *p* = 0.0002 for –DOX Basal and –DOX BaCl_2_ and ∗∗∗, *p* = 0.0002 −DOX BaCl_2_*versus* +DOX BaCl_2_). Data are shown as mean ± SD; ns, non-significant. *E*, schematic of the plate reader assay using stably expressing NPY-mApple in mock treated (−DOX) and AP-3 depleted cells (+DOX, AP-3 shRNA1). *F*, quantification of NPY secretion under basal and KCl stimulated conditions showed significantly reduced release in AP-3 depleted (+DOX) cells compared with mock treated (–DOX) cells (N = 4, Two-tailed unpaired *t* test, ∗∗∗, *p* = 0.0002, ∗∗∗∗, *p* < 0.0001). The data are shown as the mean ± SD.

To investigate the status of regulated secretion of DCVs in AP-3-depleted cells, we used a stimulus-coupled secretion experiment using the release of CgB (chromogranin B), a DCV marker (34). The cells were incubated in the basal buffer with or without BaCl_2_ (a secretagogue) for 15 min. The collected supernatants and cell homogenates were immunoblotted and probed for CgB (Fig. 2*A*). Results suggested that regulated secretion was impaired in AP-3-depleted cells (+DOX) compared to mock-treated cells (−DOX), as evidenced by lower CgB levels in cell supernatants by Immunoblotting (Fig. 2, *B* and *D*). However, in cell homogenate, the levels remained unchanged, despite a decreasing trend in mock treated (−DOX) cells under stimulation (Fig. 2*C*). These findings were validated in two other cell lines generated with different shRNAs targeting AP-3 (Fig. S1, *B* and *C*) and were similar to previous work with AP-3 depleted cells, showing a defect in the regulated secretion of secretogranin II (SgII) (21). On the contrary, prolonged basal buffer incubation showed no changes in the constitutive secretion of HSP90 protein in the supernatant as well as cell lysates (a specific constitutive marker). Similarly, the global constitutive signature was unaffected (35, 36) (Fig. S1, *D*–*G*). We also used a scrambled shRNA-mediated PC12 cell line as a control, which showed no change in CgB release (Fig. S1*H*).

In parallel, NPY (neuropeptide Y, a soluble DCV cargo) tagged with a fluorescently labelled mApple plasmid was introduced into AP-3-depleted cells to generate stable cells. Stimulus-coupled secretion experiments were subsequently performed using a buffer with or without KCl in mock-treated (−DOX) and AP-3-depleted (+DOX) cells (shRNA1), after which the fluorescence intensities in the supernatant and lysate were measured (Fig. 2*E*). The results indicated defective regulated secretion, as evidenced by reduced NPY secretion in both basal and stimulation in AP-3 depleted (+DOX) cells compared to mock-treated (−DOX) cells (Fig. 2*F*). Collectively, these experiments suggest an impeded regulated secretory pathway due to the loss of AP-3.

### Ultrastructural analysis revealed that the maturation of DCVs is compromised

**Figure 3:**
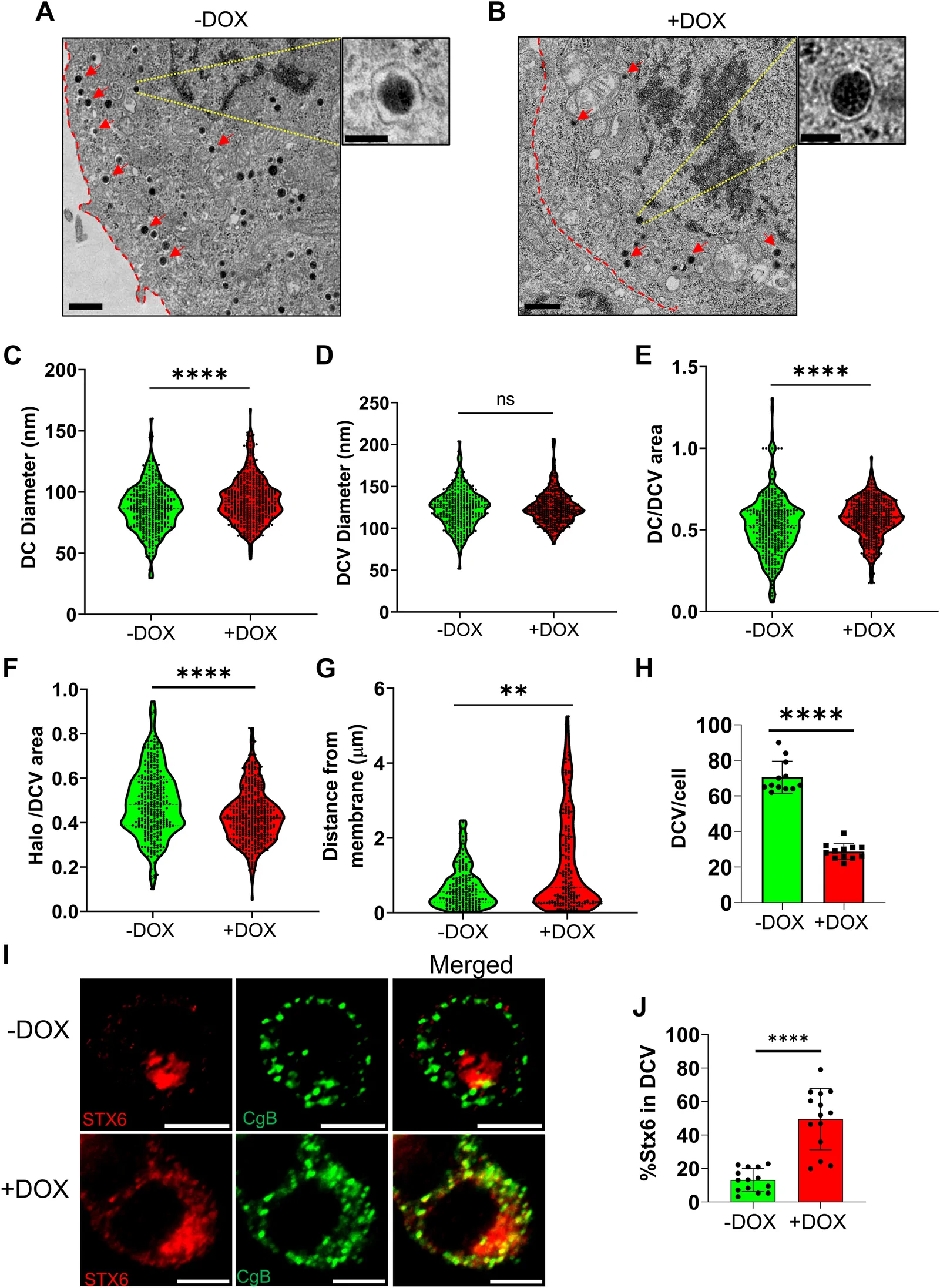
**AP-3 affects the maturation of DCVs in PC12 cells.***A–B*, representative transmission electron microscope images of mock treated (−DOX, green) and AP-3 shRNA1 depleted (+DOX, *red*) cells. Scale bar: 500 nm. Inset scale bar: 100 nm. *C*, the graph represents morphometric analysis of the dense core (DC) diameter, showing an increase in DC diameter in AP-3 depleted cells (For −DOX a total 332 DCVs were analyzed with DC diameter mean of 86.69 ± 19.01 nm, for +DOX a total 403 DCVs were analyzed with DC diameter mean of 93 ± 18.14 nm, Two-tailed Mann-Whitney U test, ∗∗∗∗, *p* < 0.0001). *D*, graph depicting analysis of the dense core vesicle diameter with no significant change (For −DOX (332 DCVs), DC diameter mean was 123.1 ± 21.87 nm, +DOX (403 DCVs) with DC diameter mean was 124 ± 18.87 nm, Two-tailed Mann-Whitney *U* test, ns, non-significant). *E*, the Graph depicts analysis of the DC area/DCV area with a significant increase in the DC area in AP-3 depleted cells (For −DOX (332 DCVs) the DC/DCV area mean was 0.5236 ± 0.1768, For +DOX (403 DCVs) the DC/DCV area mean was 0.5685 ± 0.1193, Two-tailed Mann-Whitney U test,∗∗∗∗, *p* < 0.0001). *F*, quantification for the halo area/DCV area (where the halo refers to the clear volume between the membrane and core (DCV minus DC area)) shows a significant decrease in the halo area in AP-3 depleted cells (For −DOX (332 DCVs) the Halo/DCV area mean was 0.4916 ± 0.1523 and for +DOX (403 DCVs) the Halo/DCV area mean was 0.4315 ± 0.1195, Two-tailed Mann-Whitney U test,∗∗∗∗, *p* < 0.0001). *G*, Violin plot showing an increase in the distance of DCVs from the plasma membrane upon AP-3 knockdown/depletion (KD) (For −DOX (175 DCVs) the mean distance from membrane was 0.6985 ± 0.5604 μm, for +DOX (190 DCVs) the mean distance from membrane was 1.296 ± 1.299 μm, Two-tailed Mann-Whitney U test, ∗∗, *p* = 0.0018) (*H*) Bar graph representing a significant decrease in DCV number upon AP-3 KD (N = 12 cell per condition, for −DOX mean DCV count was 70.50 ± 9.010 and for +DOX mean DCV count was 28.67 ± 4.438, Two-tailed unpaired *t* test, ∗∗∗∗, *p* < 0.0001). (Data are shown as mean ± SD). *I-J*, representative confocal images and quantification showing an increase in colocalization (object-based) represented as % colocalization) of STX6 (*red*) with CgB (DCV marker, *green*) Scale bar: 5 μm (two-tailed unpaired *t* test) (N = 3, n = 14 cells each, Data are shown as mean ± SD, ∗∗∗∗, *p* < 0.0001; ns, non-significant).

A previous report showed that decreased exocytosis can indicate possible maturation defects (22). In conjunction with a previous electron microscopy report (19), we observed a larger dense core (DC) diameter (Figs. 3, *A*–*C* and S2, *A*–*E*), whereas the diameter of the DCV remained unchanged in mock-treated (−DOX) and AP-3-depleted (+DOX) cells (Figs. 3*D* and S2*F*). Overall, upon AP-3 depletion, the DC occupied a significantly larger fraction of the DCV area (Figs. 3*E* and S2*G*) while the halo/clear fraction of DCV was reduced (Figs. 3*F* and S2*H*).

Electron microscopic images also revealed that DCVs are distantly located from the plasma membrane in AP-3 depleted cells (Figs. 3*G* and S2*I*), whereas most of these vesicles are present closer to the plasma membrane in mock-treated (−DOX) cells. Furthermore, there was a decrease in the number of DCVs (Figs. 3*H* and S2*J*) in AP-3 depleted cells. In accordance with conventional methods, an increased dense core area indicates immature vesicle formation due to a lack of functional AP-3 complexes and the accumulation of such vesicles in proximity to the TGN, resulting in an overall reduction in readily releasable pools of DCVs (19, 37).

### The increased localization of STX6 to DCVs substantiates maturation defects

A previous study revealed that STX6 is present in immature DCVs; furthermore, Clathrin/AP-1 helps remove STX6 from these vesicles, returning them to the TGN. These findings suggest that mature DCVs contain very little to no STX6 (38); therefore, STX6 can be used as a marker of immature DCVs.To further substantiate our EM experiment, we tested whether STX6 would be more localised to DCVs in AP-3 depleted cells. Hence, we compared the colocalization of STX6 with chromogranin B in mock-treated (−DOX) and AP-3-depleted (+DOX) cells. Loss of AP-3 led to a significant increase in the colocalization of STX6 with CgB (Figs. 3, *I*, *J* and S2, *K*, *L*), suggesting an accumulation of STX6 in the DCVs of AP-3-depleted cells and its defective removal from immature DCVs. Given the redundant functions of AP complexes (39, 40), AP-3 may also play a role in removing STX6 from these vesicles and assist in shuttling it back to the TGN, thus playing an additional role in DCV maturation by ‘sorting’ the cargo away from DCVs, similar to the known role of the AP-1 complex (38). However, this remains unexplored.

### The retention using selective hook (RUSH) experiments revealed slower kinetics of NPY sorting at the Golgi in AP-3 depleted cells

**Figure 4:**
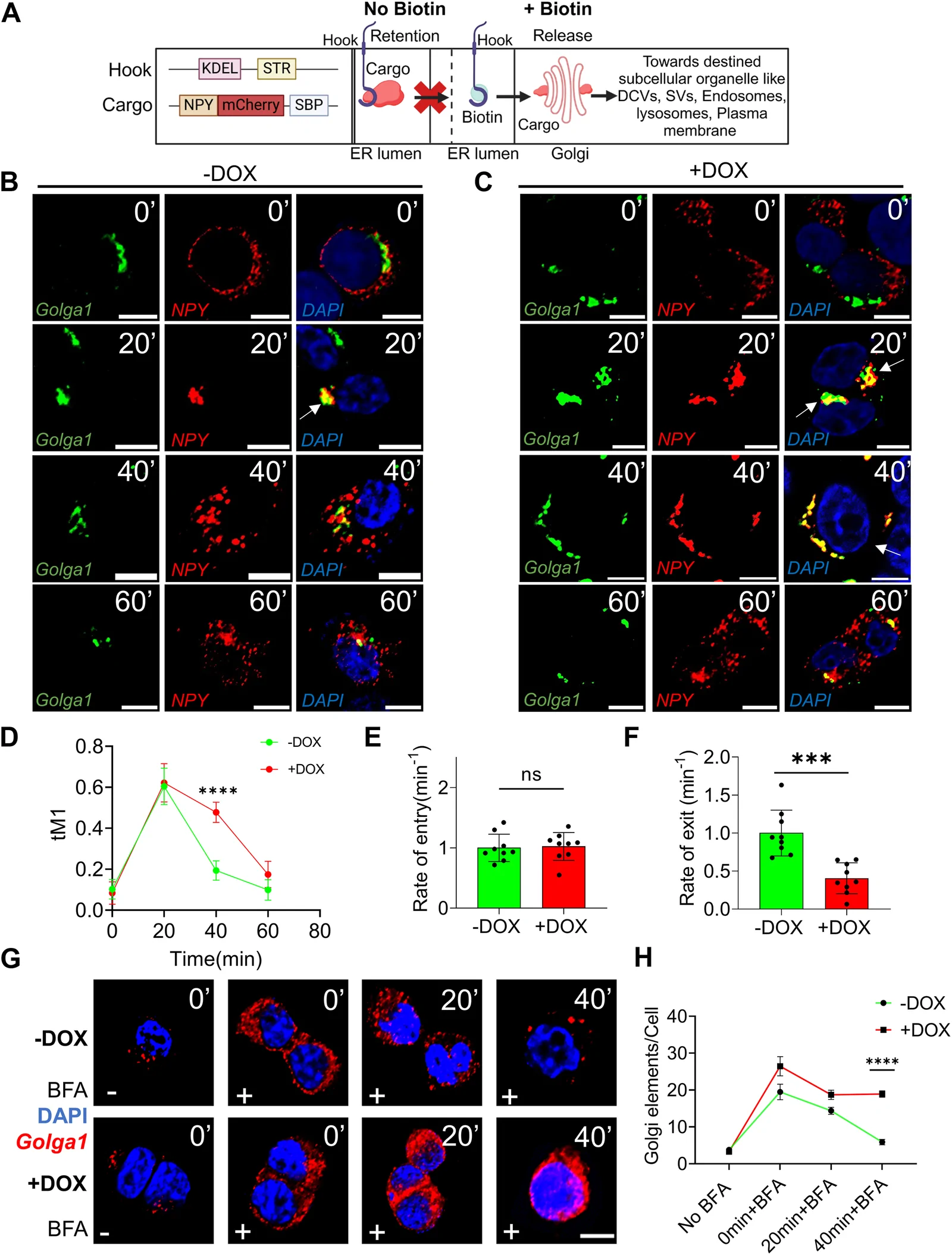
**AP3 affects the dynamic organization of the trans-Golgi network and its subsequent sorting of DCV cargo NPY.***A*, schematic of retention using a selective hook (RUSH) system to monitor trafficking of NPY cargo. *B–C*, representative confocal images of mock treated (−DOX) and shRNA1 AP-3 depleted cells (+DOX) expressing NPY-SBP-mCherry (*red*) and stained for Golga1 (*green*). Scale bar: 5 μm. *D*, quantification of colocalization between NPY and Golga1 (tM1) for mock treated (*green*, −DOX) and shRNA1 AP-3 depleted cells (*red*, +DOX) following biotin addition. A significant decrease in NPY colocalization is observed at 40 min post-biotin treatment in mock treated (−DOX) cells compared with AP-3 depleted +DOX cells. (Two-tailed unpaired *t* test, ∗∗∗∗, *p* < 0.0001) (*E*). Quantification of cargo entry into the Golgi showed no significant difference between conditions (Two-tailed unpaired *t* test, ns, non-significant). *F*, quantification of cargo exit from the Golgi demonstrated a significant reduction upon AP-3 depletion (Two-tailed unpaired *t* test, ∗∗∗, *p =* 0.002). (N = 3 independent experiments, n = 10 cells) Data are shown as mean ± SD. Scale bar: 5 μm. *G*, representative images of mock treated (−DOX) and shRNA1 AP-3 depleted cells (+DOX) treated with brefeldin A. *H*, quantification of Golgi elements per cell showing a defect in Golgi reassembly upon AP-3 KD after 40 min of brefeldin-A washout. (Multiple *t* test, correction using Holm Sidak method, ∗∗∗∗, *p <* 0.0001) (N = 3 independent experiments; n = 3 ROI per experiment). Scale bar: 5 μm. Data are shown as mean ± SD.

In addition to its contribution to DCV maturation, we sought to explore the broader spectrum of AP-3 functions at the TGN. While the journey towards mature DCVs begins post-TGN (41), AP-3-dependent sorting of cargoes to DCVs at the TGN is poorly understood. Hence, we used a retention-using-selective-hook (42) approach to track the transport of a soluble DCV cargo, NPY, from the endoplasmic reticulum (ER) through the Golgi to DCVs (Fig. 4*A*) (43). In the absence of biotin, NPY is localized throughout the cytoplasm, consistent with ER retention due to the KDEL motif. The addition of biotin released NPY from the ER hook, allowing synchronous trafficking toward the Golgi. In both mock-treated (−DOX) and AP-3-depleted (+DOX) cells, NPY accumulated at the Golgi within 20 min and showed a near-complete colocalization with the TGN marker Golga 1 (Golgin 97), indicating typical ER to Golgi transport (Fig. 4, *B* and *D*).

Following entry into the Golgi, NPY progressively exited the Golgi in mock-treated (−DOX) cells, with most cargo sorted into post-Golgi vesicles by 40 min and nearly complete clearance from the TGN by 60 min (Fig. 4, *B* and *D*). In contrast, AP-3 depletion resulted in significantly greater NPY retention at the TGN at 40 min, indicating delayed cargo sorting from the Golgi (Fig. 4, *C* and *D*). Although NPY eventually exited the TGN by 60 min, this transient accumulation suggested a defect in the DCV cargo sorting at the TGN. Consistent with this, kinetic analysis revealed no change in the rate of NPY entry into the Golgi but a significant reduction in the rate of NPY exit from the TGN upon AP-3 depletion (Fig. 4, *E* and *F*). Similar observations were obtained using an independent shRNA2-mediated AP-3 depletion (Fig. S3, *A*–*E*), highlighting the role of AP-3 in the sorting of DCVs at the TGN.

### Brefeldin A experiments suggested a dysfunctional Golgi

AP-3 is a coat protein that is dependent on ARF1 and the Arf-GAP AGAP1 for its binding with endolysosomal membranes, where ARF1 inhibition by brefeldin A (BFA) leads to Golgi disassembly (13, 44). Therefore, we sought to visualize the dynamic organization of the Golgi and to determine whether AP-3 is involved in Golgi function. A brief incubation with BFA (15 min) disrupted the Golgi, which began to reassemble after BFA washout in mock-treated (−DOX) cells, as assessed by staining with the TGN marker Golgin 97. At 40 min post-washout of BFA in mock-treated (−DOX) cells, the Golgi elements per cell decreased, suggesting overall reassembly of the organelle (Figs. 4, *G*, *H* and S3*F*).

Interestingly, in AP-3 depleted cells, Golgi disassembly was irreversible; *i.e.*, after washing out the BFA, no significant reduction in Golgi fragmentation was observed (Figs. 4, *G*, *H* and S3*F*). A similar defect in the reassembly of the Golgi apparatus was previously reported in the case of the loss of the Clathrin heavy chain (45). This may suggest that AP-3, along with the Clathrin heavy chain, also plays an important role in the reorganization of the Golgi, and that dysfunction of the AP-3 complex can disrupt sorting sites and cause missorting of cargo loaded onto DCVs, which may be retrieved *via* recycling.

### Specific DCV cargo is depleted in the vesicle-enriched fractions (VEFs) of AP-3-perturbed cells

**Figure 5:**
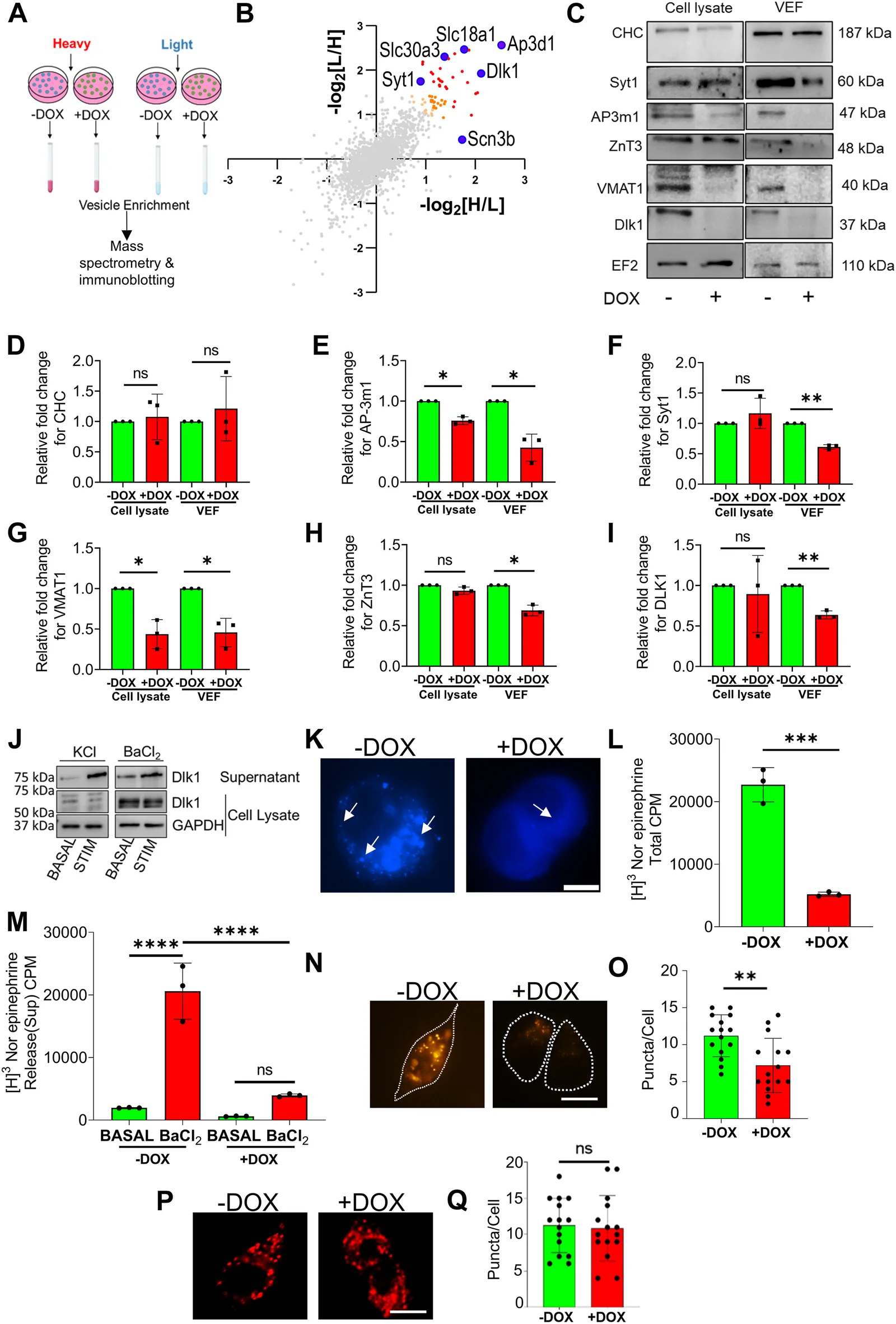
**AP-3 plays a selective role in sorting membrane proteins and affects the trafficking of vesicles post-Golgi.***A*, schematic of the SILAC-based mass spectrometry approach for assessing VEFs in mock treated (−DOX) and shRNA1 AP-3 depleted PC12 cells (+DOX). *B*, scatter plots against -log2[H/L] and -log2[L/H] in 2 separate experiments showing deficient proteins in VEFs (colored dots represent significantly depleted proteins) for mock treated (−DOX) *versus* shRNA1 AP-3 depleted cells, including known and candidate DCV cargoes. *C*, WB validation of selected in the cell homogenate and VEFs under –DOX and +DOX conditions. (*D*) Quantification of fold change for CHC in both cell lysates and CCV fractions remains unchanged (n = 3, Two-tailed unpaired *t* test with Welch’s correction, ns, non-significant). *E*, quantification of fold change for AP-3m1 in both cell lysates and CCV fractions was reduced (n = 3, Two-tailed unpaired *t* test with Welch’s correction, ∗, *p* = 0.0129, ∗, *p* = 0.0271 respectively). *F*, quantification of fold change for SYT1 in CCV fraction shows a significant decrease while the levels remain unaffected in cell lysates (n = 3, Two-tailed unpaired *t* test with Welch’s correction, ∗∗, *p* = 0.003, ns, non-significant). *G*, quantification of fold change for VMAT-1 in both cell lysates and CCV fractions was reduced (n = 3, Two-tailed unpaired *t* test with Welch’s correction, ∗, *p* = 0.0323 and *p* = 0.0332 respectively). *H*, quantification of fold change for ZnT3 in CCV fraction shows a significant decrease while the levels remain unaffected in cell lysates (n = 3, Two-tailed unpaired *t* test with Welch’s correction, ns, non-significant, ∗, *p* = 0.0143). *I*, quantification of fold change for DLK1 in CCV fraction shows a significant decrease while the levels remain unaffected in cell lysates (n = 3, Two-tailed unpaired *t* test with Welch’s correction, ∗∗, *p* = 0.0062, ns, non-significant). *J*, Western blot representation of DLK1 in WT PC12 cells incubated with BaCl_2_ for 15 min and KCl for 20 min, examining both the cells and supernatants. *K*, representative fluorescence images of mock treated (−DOX) and shRNA-1 AP-3 depleted cells (+DOX) showing reduced Zinquin ethyl ester dye uptake. Scale bar: 5 μm. *L*, the graph depicts approximately four-fold greater uptake of nor-epinephrine in the mock treated (−DOX) cells after adding the counts in both the cells and the medium. (N = 3, two-tailed unpaired *t* test, ∗∗∗, *p* = 0.0004). *M*, in mock treated (−DOX) cells, release of [^3^H] norepinephrine was strongly dependent on BaCl_2_ stimulation, but in the AP-3 depleted cells (+DOX), the effect of BaCl_2_ was insignificant. (N = 3, independent experiment, Data shown as mean ± SD, ∗∗∗∗*p* < 0.0001 level assessed by analysis of variance with Tukey’s *post hoc* test; ns, non-significant). *N–O*, representative images and quantification of Dextran 40 kDa uptake showing impaired expanision of larger fusion pores, as the puncta per cell are reduced in shRNA1 AP-3 depleted cells (*red*, +DOX) compared with mock treated cells (*green*, −DOX). (N = 3, n = 15 cells each, two-tailed unpaired *t* test, ∗∗, *p* = 0.0024). (*P*–*Q*) Representative images and quantification of Dextran 10 kDa uptake show no significant changes in mock treated (*green*, −DOX) *versus* shRNA1 AP-3 depleted cells (*red*, +DOX). (N = 3. N = 15 cells each, two-tailed unpaired *t* test, ns, non-significant). Data are shown as mean ± SD.

AP-3 has been implicated in the sorting of various cargo proteins, such as zinc transporter-3 (ZnT-3) and chloride channel (ClC-3), which have been reported as signature proteins of both DCVs and synaptic-like vesicles (SLVs) (46, 47). However, whether AP-3 also sorts distinct or novel cargo specific to each compartment remains poorly understood. For this purpose, we used a comparative proteomics approach to identify the various cargo and other accessory proteins associated with AP-3. Mock treated (−DOX) and AP-3 depleted (+DOX) cells were grown in “light” and “heavy” stable isotopes of amino acids (arginine and lysine) for 3 weeks. Incorporation of isotopically distinct amino acids enables stable isotope labelling by amino acids in cell culture (SILAC)-based quantitative proteomics, allowing peptides from control and experimental samples to be distinguished by their characteristic mass shift in the mass spectrometer while remaining chemically identical. This strategy permits accurate, multiplexed, and internally normalized quantification of relative protein abundance between conditions by mass spectrometry (22). After labelling, the cell lysates were fractionated *via* differential ultracentrifugation to obtain a vesicle-enriched fraction comprising mainly clathrin-coated vesicles, dense-core vesicles, and synaptic-like vesicles (Fig. 5*A*, see methods). This method and analysis were based on previous works (22, 48). From the mass spectrometric analysis, we detected approximately 2000 proteins, including soluble and membrane proteins of DCVs, represented as scatter plots against -log2[H/L] values showing deficient proteins in VEFs (colored dots represent significantly depleted proteins) for mock-treated (−DOX) *versus* shRNA1 AP-3-depleted (+DOX) cells (Fig. 5*B*). Clathrin heavy chain (CHC) is the most abundant protein in the vesicle-enriched fraction and served as a control for this fractionation experiment (48). CHC was enriched in the fractions under unaltered mock-treated (−DOX) and AP-3-depleted (+DOX) conditions. AP-3 can function in both clathrin-dependent and clathrin-independent manners, which explains the unaffected protein level of Clathrin heavy chain (41, 49) (Figs. 5, *C*, *D* and S4*A*). Sixty-six proteins were significantly reduced in the VEFs of AP-3-depleted (+DOX) cells compared with mock-treated (−DOX) cells (Table S1).

Some of these candidates were validated by western blotting, based on antibody availability (Figs. 5, *C*–*I* and S4*A*). AP-3 complex subunits, such as AP-3 β and σ, have also been reported to be depleted, whereas the μ subunit was detected only in one run which showed reduced levels both in cell lysates and vesicular fraction (Fig. 5*E*). Among the significantly depleted proteins, we report the presence of multiple ion channels (VDAC1, SCN3B) and transmembrane proteins (DLK1, VMAT1, ZnT3, SYT1), which may depend on the AP-3 complex for sorting. Specifically, we highlight Synaptotagmin-1 (SYT1), a calcium sensor that facilitates vesicle fusion and neurotransmitter release from DCVs (26), which was reduced in the AP-3 depleted vesicle fraction (Figs. 5*F* and S4*A*). Moreover, its levels in the cell homogenate remained unchanged in AP-3-depleted shRNA1 cells, a finding consistent with previous reports (Fig. 5*F*) (21); however, we did observe a reduction in SYT1 in cell homogenates in shRNA2-mediated knockdown. We speculate that shRNA2 is more efficient at depleting the AP-3 complex and therefore observe a reduction in SYT1 levels in the cell homogenate as well. Similarly, vesicular monoamine transporter 1 (VMAT1), another DCV cargo responsible for the loading of catecholamines into DCVs (27), was markedly depleted from the vesicular fraction as well as whole-cell homogenate in the absence of AP-3 (Figs. 5*G* and S4*A*). This finding suggests the possible involvement of AP-3 in sorting VMAT1 toward the regulated secretory pathway. We also reported that the abundance of ZnT3 in the vesicular fractions was reduced with unhindered presence in cell lysates (Figs. 5*H* and S4*A*), which is consistent with previous reports (46). Interestingly, we report that Delta-like homologue 1 (DLK1), a noncanonical notch ligand‒receptor, also diminished from vesicular fractions while remaining unaffected in cell lysates (Fig. 5*I*). Notably, the absence of DLK1 transcripts in a clonal PC12 cell line resulted in defective regulated secretion (50). Here, we report that DLK1 is a novel DCV cargo that is secreted in a regulated manner based on the secretagogue-based release of DLK1 upon stimulation (Fig. 5*J*). Given the clear loss of DLK1 in the absence of AP-3, we suggest that DLK1 is a novel AP-3 dependent DCV cargo. Similarly, DLK1 was not present on synaptic-like vesicles (Fig. S4*B*), implying that DLK1 is specific to DCVs.

### The mislocalization of ZnT3 and VMAT1 is consistent with functional defects in the accumulation of neurotransmitters and zinc in the DCV compartment

Based on proteomics data, we examined the functions of proteins depleted from the vesicle-enriched fractions. The function associated with ZnT3 is the accumulation of zinc inside SLVs and DCVs (46, 51, 52). Therefore, we used Zinquin ethyl ester (ZEE) (53), a dye that specifically binds to the zinc ions inside the vesicles. Mock treated (−DOX) cells incubated with zinc ions and stained with ZEE presented distinct punctate staining, indicating zinc uptake and mobilization, whereas AP-3 depleted (+DOX) cells did not show any puncta, indicating defective uptake of zinc (Figs. 5*K* and S4*C*). The disappearance of ZnT3 due to AP-3 depletion possibly accounts for this defect. The uptake and release of catecholamines (norepinephrine, epinephrine, dopamine) depend on VMAT1, which is primarily found on regulated secretory vesicles. Therefore, we investigated norepinephrine secretion *via* a catecholamine uptake assay (22) (see methods). The cells were first incubated in a medium containing [3H] norepinephrine for 3 h. Post which, the cells were washed and incubated for 15 min with or without BaCl_2_. The radioactivity measured from the collected cell lysates and supernatants was summed to yield a measure of total [3H] norepinephrine uptake for each condition. This finding revealed that AP-3 loss led to a four-fold reduction in nor-epinephrine uptake compared with mock treated (−DOX) cells (Figs. 5*L* and S4*D*). In the mock treated (−DOX) cells, the release of norepinephrine was strongly dependent on the BaCl_2_ stimulated depolarization, as there was a significant increase in the release of norepinephrine in the presence of BaCl_2_ in the supernatants (Figs. 5*M* and S4*E*). While, in the AP-3 depleted (+DOX) cells, the release was insignificant when the cells were incubated in the presence of BaCl_2_ in the supernatants (Figs. 5*M* and S4*E*). Thus, AP-3 depletion causes an impaired stimulus-coupled catecholamine release. This finding again corroborates the finding of reduced VMAT1 expression in the AP-3 depleted vesicle-enriched fractions. Hence, when AP-3 is depleted from these cells, the localization of ZnT3 and VMAT1 may be lost, indicating a sorting defect. AP-3 is also localized at early and recycling endosomes, where it regulates the trafficking of cargoes to lysosomes or lysosome-related organelles (54). Hence, we validated defective endolysosomal function upon AP-3 depletion using 10 kDa dextran (as described in the methods section), which resulted in delayed clearance of puncta at 30 min and 60 min after dextran uptake (Fig. S4, *H* and *I*). Thus, this signifies the importance of AP-3 in regulating exocytotic recycling and clearance *via* the endolysosomal pathway.

### Depletion of SYT1, a key protein of the SNARE complex, corresponds to defective dilation of fusion pores

There are multiple modes of regulated exocytosis based on the fusion pore dynamics: kiss-and-run involves transient fusion events of the secretory granule with the plasma membrane associated with restricted opening of the fusion pore, cavicapture events where transient fusion of secretory granules undergoes dilation of the fusion pore allowing partial release of larger neuropeptides while keeping the omega shape of the fusing vesicle preserved, and full fusion events characterized by complete fusion of a single secretory granule with the plasma membrane (55, 56). The extent of fusion pore opening influences the entry/exit of molecules into secretory granules during stimulation. Fluorescent dextrans of different molecular sizes have been used as probes of fusion pore permeability. Smaller dextrans (10 kDa) can enter through relatively narrow fusion pores, whereas uptake of the larger dextrans (40 kDa) requires formation of larger pore openings (57, 58, 59, 60). This assay provides bulk measurements of fusion pore permeability while it does not definitively resolve individual modes of exocytosis.

To determine whether AP-3 depletion alters fusion pore properties during regulated exocytosis, mock-treated (−DOX) and AP-3 depleted (+DOX) cells were stimulated with a buffer containing a high potassium concentration in the presence of either 40 kDa or 10 kDa rhodamine dextran for 1 min. AP-3 depletion led to a decrease in the uptake of 40 kDa Dextran, whereas the uptake of 10 kDa dextran was not altered (Figs. 5, *N*–*Q* and S4, *F*, *G*). These findings suggest a preferential impairment of exocytotic events associated with the formation of larger pore openings, while preserving the narrow pore opening that permits the entry of smaller molecules. This observation is consistent with SYT1 depletion in the AP-3-deficient VEF, as evidenced by our proteomic analysis, as SYT1 functions as a prominent Ca2+ sensor that critically regulates the final stage of vesicle fusion with the plasma membrane while also helping to dilate the fusion pores (61). The dextran uptake assay indicates alterations in fusion pore dilation of secretory granules but with limited resolution. In our experiments, AP-3 depletion indicates defective fusion pore dilation, contributing to the secretory defect.

### Reduced localization of SYT1, VMAT1 and DLK1 with the DCV compartment

**Figure 6:**
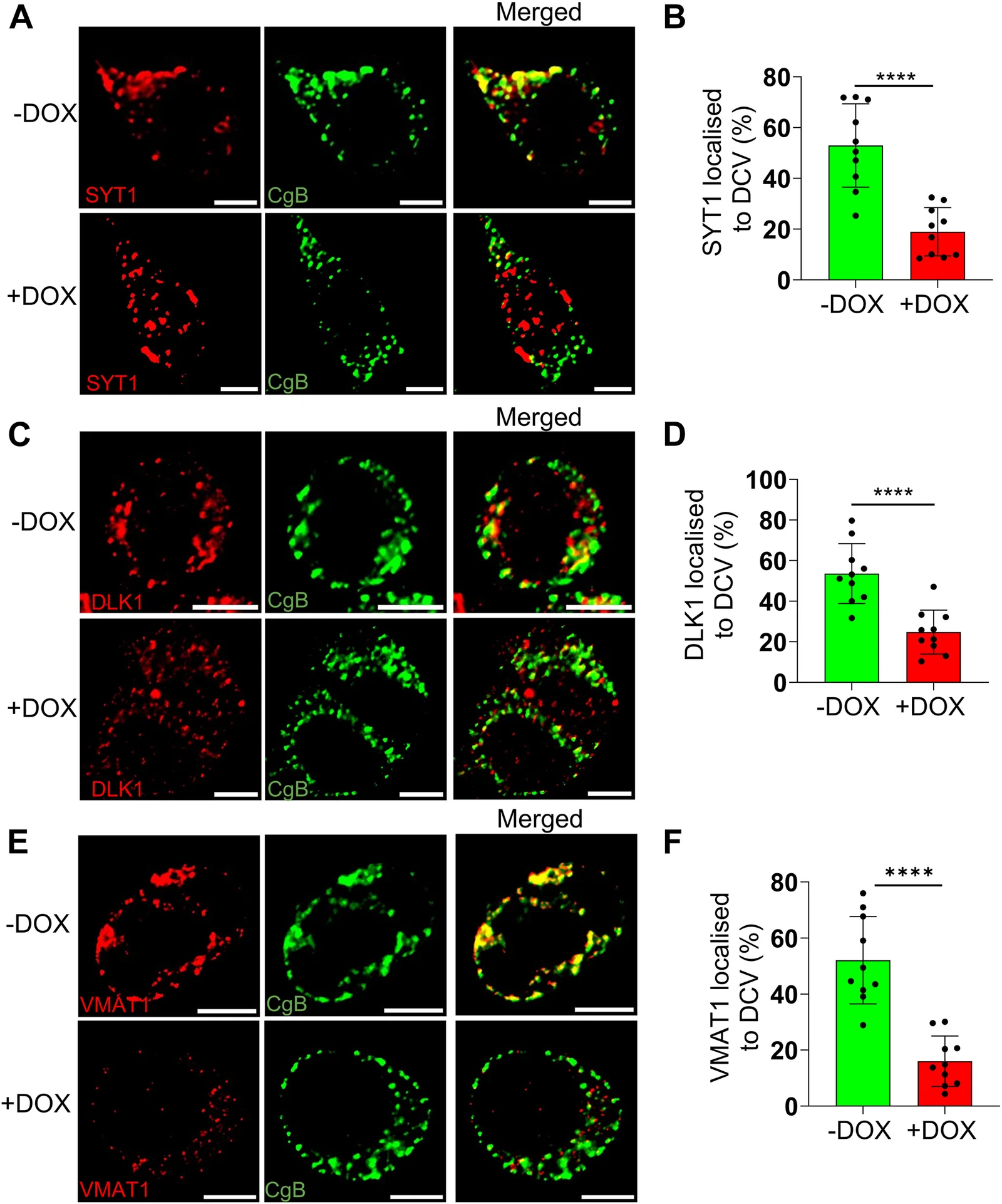
**AP-3 preferentially sorts specific membrane proteins mislocalized during the loss of AP-3 in PC12 cells.***A–B*, Representative confocal images and graph showing a decrease in colocalization (tM1 represented as % colocalization) of SYT1-mcherry (*red*) with the DCV marker CgB (*green*) in shRNA1 AP-3 depleted cells (+DOX) compared to mock treated (−DOX) cells. (n = 10 cells from N = 3 independent experiments, two-tailed unpaired *t* test, ∗∗∗∗*p* < 0.0001). *C–D*, representative confocal images and graph showing a decrease in colocalization (tM1 represented as % colocalization) of DLK1 (*red*) with the DCV marker CgB (*green*) in shRNA1 AP-3 depleted cells (+DOX) compared to mock treated (−DOX) cells. (n = 10 cells from N = 3 independent experiments, two-tailed unpaired *t* test, ∗∗∗∗*p* < 0.0001). *E-F*, representative confocal images and graph showing a decrease in colocalization (tM1 represented as % colocalization) of VMAT1 (*red*) with the DCV marker CgB (*green*) in shRNA1 AP-3 depleted cells (+DOX) compared to mock treated (−DOX) cells. (n = 10 cells from N = 3 independent experiments, two-tailed unpaired *t* test, ∗∗∗∗*p* < 0.0001). Data are shown as mean ± SD.

We have shown that the lack of the AP-3 protein disrupts the transport of secretory vesicles in addition to a defective exocytosis. This is substantiated by the loss of key signature proteins, such as SYT1, VMAT1, and ZnT-3, which are known to be essential for vesicle function. We hypothesised that these proteins may be missorted and, to test this, employed confocal microscopy to assess their colocalization, which was significantly decreased. We examined the localisation of SYT1, DLK1, and VMAT1 using the DCV marker CgB in mock-treated (−DOX) and AP-3-depleted (+DOX) cells. Confocal microscopy revealed near-complete loss of colocalization of SYT1, DLK1, and VMAT1 with CgB in AP-3 depleted cells (Figs. 6, *A*–*F* and S5, *A*–*F*). These findings suggest that AP-3 is essential for sorting these known signature proteins into DCVs. To comprehensively evaluate the impact of AP-3 depletion on DCV cargo localization, we further investigated proteins identified by mass spectrometry as unaffected by AP-3 knockdown. We assessed the colocalization of NPY soluble cargo, phogrin, Synaptotagmin 4 (SYT4), Synaptotagmin 7 (SYT7), and Vesicular monoamine transporter 2 (VMAT2) with the DCV marker CgB in both mock-treated (−DOX) and shRNA1-mediated AP-3-depleted (+DOX) cells. Our findings revealed no significant difference in the colocalization patterns of these signature proteins between mock-treated and AP-3-depleted cells (Fig. S6), supporting our hypothesis that AP-3 preferentially sorts specific cargo molecules destined for DCVs.

### Augmented localization of SYT1, VMAT1 and DLK1 to lysosomes

Lysosomes act as the main recycling centers of cells, breaking down proteins that have reached their lifespan, are improperly folded, or are improperly delivered within the cell. To further determine the fate of mislocalized proteins due to AP-3 loss of function, we investigated the final destination of these cargo proteins by colocalizing them with a lysosomal marker (lyso 20 or LAMP1) in both mock-treated (−DOX) and AP-3-depleted (+DOX) cells.

**Figure 7:**
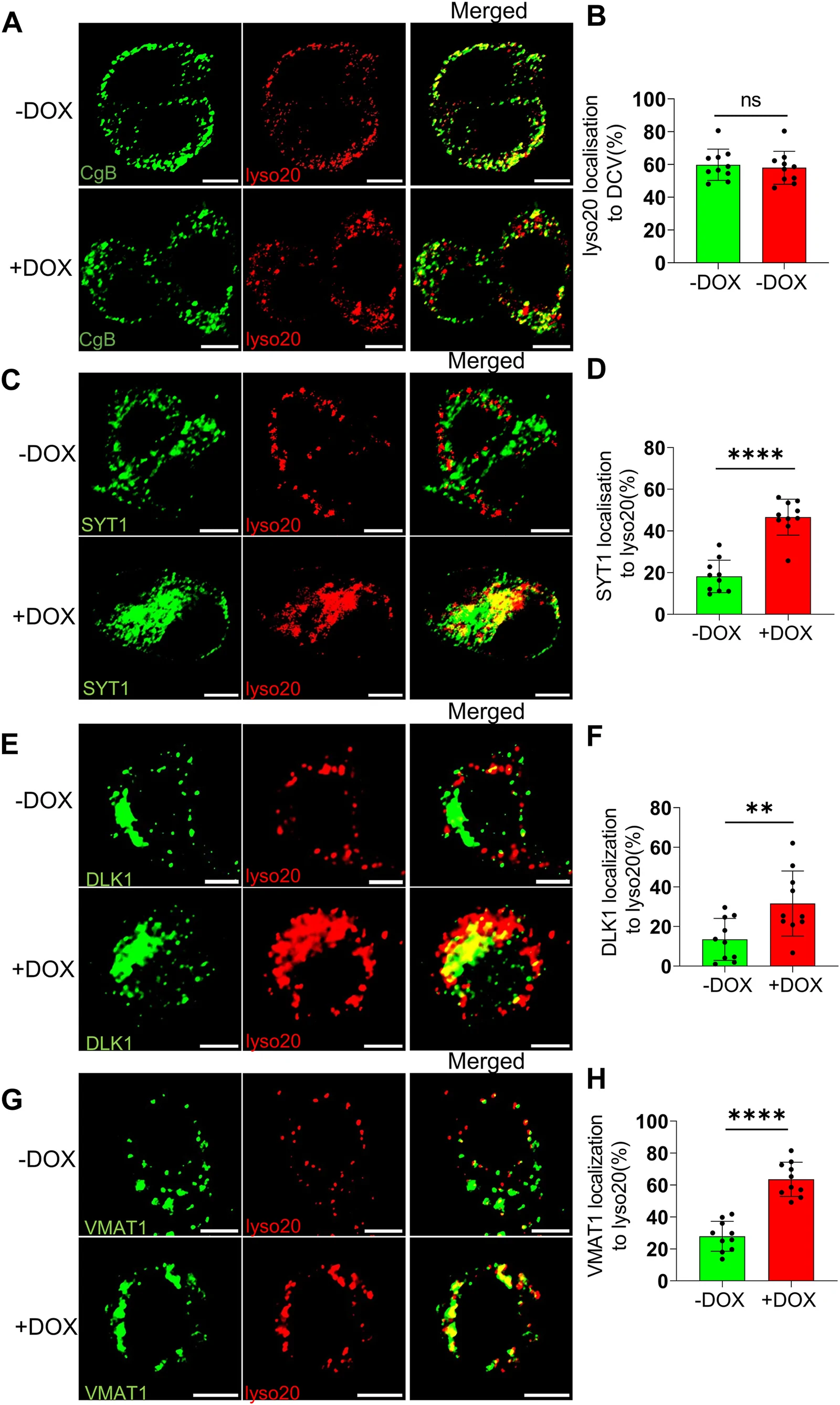
**Missorted proteins are rerouted to lysosomes.***A–B*, representative images and graph showing no change in the colocalization (tM1 represented as % colocalization) of the DCV marker CgB (*green*) with the lysosomal marker Lyso20-mCherry (LAMP1) (*red*) in mock treated (−DOX) and AP-3 depleted cells (+DOX) (n = 10 cells from N = 3 independent experiments, two-tailed unpaired *t* test, ns, non-significant). *C–D*, representative images and graph showing an increase in the colocalization (tM1 represented as % colocalization) of the SYT1 (*green*) with the lysosomal marker Lyso20-mCherry (LAMP1) (*red*) in AP-3 depleted cells (+DOX) compared to mock treated (−DOX) cells (n = 10 cells from N = 3 independent experiments, two-tailed unpaired *t* test, ∗∗∗∗, *p* < 0.0001). *E-F*, representative images and graph showing an increase in the colocalization (tM1 represented as % colocalization) of the DLK1 (*green*) with the lysosomal marker Lyso20-mCherry (LAMP1) (*red*) in AP-3 depleted cells (+DOX) compared to mock treated (−DOX) cells (n = 10 cells from N = 3 independent experiments, two-tailed unpaired *t* test, *p* = 0.0091). *G-H*, Representative images and graph showing an increase in the colocalization (tM1 represented as % colocalization) of the VMAT1-mCherry (*red*) with the lysosomal marker Lyso20-BFP (LAMP1) (*green*) in AP-3 depleted cells (+DOX) compared to mock-treated (−DOX) cells (n = 10 cells from N = 3 independent experiments, two-tailed unpaired *t* test, ∗∗∗∗, *p* < 0.0001). Scale bar: 5 μm. Data shown as mean ± SD, ∗*p* < 0.05, ∗∗*p* < 0.01, ∗∗∗*p* < 0.001, ∗∗∗∗*p* < 0.0001; ns, non-significant.

AP-3 knockdown significantly increased the colocalization of SYT1, DLK1, and VMAT1 with lysosomal markers (Figs. 7, *C*–*H* and S7, *C*–*H*). This approach revealed potential misrouting of the cargo proteins towards lysosomes upon AP-3 knockdown. Unlike SYT1, DLK1, and VMAT1, the localization of CgB (a marker for DCVs) remained unchanged relative to that of lysosomes in AP-3 depleted cells (Figs. 7, *A*, *B* and S7, *A*, *B*). These findings suggest that AP-3 deficiency does not affect the sorting of soluble cargo such as CgB. Our findings suggest that AP-3 is critical for targeting specific membrane proteins (SYT1, DLK1, and VMAT1) to DCVs. This may explain the reduction in these proteins in VEFs upon AP-3 depletion, resulting from their mis-sorting from DCVs to lysosomes.

### Computational analysis *via* Colabfold2 predicts physical associations of AP-3 with SYT1 and VMAT1

Loss of specific DCV signature proteins in the VEFs of AP-3-depleted cells was accompanied by missorting and rerouting of these proteins to lysosomes, establishing the role of the AP-3 complex in sorting them into DCVs. We were curious to investigate whether there are any physical interactions among these proteins with the AP-3 complex. This prompted us to use computational biophysics and bioinformatics to predict interactions among these proteins using the recently developed platform ColabFold (62). We examined candidate proteins that had been mislocalized in microscopy experiments and were lost in VEFs. The AlphaFold model compared all subunits of the AP-3 complex with the SYT1, VMAT1, and DLK1 protein sequences (Fig. S8*A*). The predicted local distance difference test (pLDDT) values above 70 correspond to a near-native structure prediction (63, 64). The Predicted Template modelling (pTM) score and interfacial pTM (ipTM) threshold were set to 0.3 or higher (65). Thes39e parameters, along with a high predicted aligned error (PAE) score, suggested possible interactions in our case. We report that SYT1 interacts with the AP-3μ1 subunit with a pTM of 0.439, an ipTM of 0.425, and a high mean pLDDT of 73.7 (Fig. S8*B*). Overall, a higher PAE score indicates a possible interaction between SYT1 and the AP-3μ1 subunit *via* a tyrosine motif (YNSTG) at the C-terminus of SYT1. Moreover, we identified a di-leucine motif, EEKRAIL, at the C-terminus of VMAT1, which is now predicted to interact with AP-3σ1, with a pLDDT of 73.2, pTM of 0.63 and ipTM of 0.66, along with a higher PAE score (Fig. S8*C*). DLK1 also showed a promising interaction with the AP-3σ2 subunit at the C-terminus of DLK1 based on its high PAE score (Fig. S8*D*). The tyrosine and di-leucine sorting motifs are important for directing cargoes to endosomal and lysosomal compartments from the TGN and/or the endosome (11, 66, 67). These motifs have also been implicated in cargo sorting *via* the AP-1 complex, mediated by the σ and μ subunits, similar to what we have reported for AP-3 (68).

These interactions were validated *in vivo* in PC12 cells *via* a proximity ligation assay (69) with SYT1, VMAT1 (in which wild-type PC12 cells overexpressing VMAT1 GFP were probed with a GFP Ab), DLK1, and AP-3δ, which further presented a positive PLA signal, indicating a possible interaction between these cargoes and the AP-3 complex (Fig. S8, *E*–*H*). Additionally, we have also performed coimmunoprecipitation experiments, further substantiating these physical associations with the AP-3 complex. In PC12 cells, the AP-3μ subunit was successfully coimmunoprecipitated with the cargo VMAT1 (Fig. S8*I*). Ultimately, AP-3 could be attributed to physical interactions with preferentially sorted DCV membrane cargoes, DLK1, VMAT1, and SYT1. Moreover, depletion of AP-3 allows these cargoes to be rerouted to lysosomes for degradation, leading to overall disruption of membrane trafficking functions and defective regulated exocytosis.

## Discussion

Dense core vesicles (DCVs) are secretory organelles specialized for the regulated release of neuropeptides, neuromodulators, and hormones. These vesicles require selective cargo sorting at the TGN, followed by vesicle maturation through cargo condensation and homotypic fusion, ultimately leading to stimulus-coupled regulated exocytosis at the plasma membrane (70, 71, 72). While adaptor protein complexes are recognized for their roles in regulating vesicle formation and cargo selection throughout the secretory pathway, their specific contribution to the coordinated maturation and functional competence of DCVs remains incompletely elucidated. This study identifies the adaptor complex AP-3 as a significant regulator of DCV trafficking and maturation. By integrating exocytotic defects in inducible knockdown of AP-3 in PC12 cells, our results indicate that AP-3 participates in multiple stages of the DCV lifecycle, including cargo transport from the Golgi apparatus, vesicle maturation, and efficient stimulus-dependent secretion.

Adaptor complexes regulate vesicle budding by orchestrating the recruitment of both cargo proteins and the coat protein machinery, which are crucial for establishing vesicle identity (1, 7). Previous studies involving AP-3 have demonstrated its participation in the biogenesis of lysosome-related organelles, specialized secretory organelles, and related trafficking pathways (9, 73, 74). The present study extends these observations by demonstrating the role of AP-3 in DCV maturation in neurosecretory PC12 cells. The maturation of immature DCVs into mature forms is a complex, multistep process that encompasses the gradual condensation of luminal cargo, the expulsion of proteins not destined for DCVs *via* clathrin-mediated budding, and the homotypic fusion of immature vesicles (70, 71). Disruptions in these stages result in observable changes in vesicle morphology and reduced secretory capacity (37). Consequently, the ultrastructural alterations observed following AP-3 depletion, such as enlarged dense-core diameter, reduced halo area, and DCVs distributed farther from the plasma membrane, are consistent with a compromised DCV maturation pathway. These findings argue against a simple hypothesis, where secretory defects are attributed exclusively to a cargo deficiency, instead indicating that AP-3 plays a role in maintaining the cellular mechanisms essential for appropriate vesicle maturation.

The process by which AP-3 influences DCV maturation likely involves regulating membrane trafficking pathways at the junction between the Golgi apparatus and endosomes. A potential component of this pathway is STX6, a SNARE protein shown to facilitate the homotypic fusion of immature secretory vesicles during DCV maturation (75). Consequently, the proper retrograde transport of STX6 back to the TGN is critical for ensuring that immature vesicles retain their capacity for fusion. Our findings indicate that AP-3 contributes to this process by facilitating the retrograde trafficking of STX6, which is required to sustain its proper subcellular localization. Prior research has established that clathrin and the adaptor complex AP-1 regulate the trafficking of STX6 and other Golgi-resident SNAREs (38, 75), implying that a coordinated action among multiple adaptor complexes is necessary to maintain the distribution of SNAREs within the Golgi-endosomal system. The concept of functional redundancy among adaptor complexes has been proposed as a strategy to ensure the adaptability of intracellular transport mechanisms (39, 40). In this framework, AP-3 might operate in conjunction with AP-1-mediated pathways to validate the proper recycling of the fusion machinery, which is indispensable for DCV maturation.

The involvement of AP-3 in regulating the trafficking of DCVs might also indicate broader roles in maintaining Golgi organization and cargo sorting. The structural organization of the Golgi apparatus is maintained by a dynamic equilibrium between anterograde and retrograde transport pathways, which are essential for the proper distribution of resident proteins and cargo receptors (76). Any disruption of vesicle budding or recycling mechanisms can therefore compromise Golgi organization and lead to aberrant cargo transport. Consistent with this concept, defects in Golgi reassembly after brefeldin A removal have been observed when components of vesicle coats, such as clathrin, are perturbed (45). Our findings suggest that AP-3 could contribute to these processes by facilitating retrograde trafficking pathways that maintain the TGN sorting domains. Impairment of these pathways could diminish the efficiency of DCV cargo exit from the Golgi. Consistent with this interpretation, analysis of NPY cargo trafficking dynamics reveals that the absence of AP-3 causes delayed exit of NPY from the TGN. Such kinetic defects in cargo trafficking can alter the composition and maturation of DCVs, ultimately influencing the efficiency of regulated secretion.

Effective regulated secretion requires not only mature vesicles but also specific membrane proteins that mediate vesicle fusion with the plasma membrane. SYT-1 functions as the primary Ca^2+^ sensor that triggers rapid vesicle fusion during regulated exocytosis (77). Our functional and proteomic data indicate that AP-3 depletion specifically disrupts the exocytic machinery, consistent with the depletion of SYT1 and other DCV-associated proteins. The dextran uptake assay has limited resolution and does not unequivocally distinguish the distinct modes of vesicle fusion; rather, it could be interpreted as evidence for fusion pore expansion. The 40-kDa dextran uptake, dependent on larger fusion pore opening, exhibited decreased uptake in AP-3-deficient cells, while the uptake of 10-kDa dextran (uptake is dependent on narrow fusion pore opening) was mostly unaffected. This distinction indicates that transient fusion events with restricted pore sizes are largely preserved, whereas the ability of the fusion pore to dilate sufficiently to permit the passage of larger molecules is impaired upon AP-3 depletion. Efficient SNARE complex formation and membrane zippering are necessary for proper dilation of the fusion pore; partial disruption of SYT1 and SNARE complexes might specifically affect luminal cargo release by hindering expansion of fusion pores, thereby influencing full-fusion events (61, 78, 79). These findings imply that AP-3 depletion, in turn, affects fusion pore expansion, limiting the passage of larger DCV cargo molecules/neuropeptides and contributing to the overall secretory defect.

The reduced abundance of SYT-1 and VMAT1, as observed in vesicle-enriched fractions post-AP-3 depletion, and the increase in colocalization with lysosomes indicate that AP-3 plays a role in the proper sorting of these proteins into DCVs. Previous reports had shown reduced SYT1 levels in DCVs in cells lacking the AP-3 complex (21). These findings serve as an independent validation of reduced abundance and support its dependence on AP-3-mediated trafficking to the DCVs. The AlphaFold modelling predicted a physical association of SYT1 specifically with the μ1 subunit of AP-3 *via* a tyrosine motif (11), and PLA further showed proximity between SYT1 and the AP-3 complex (δ subunit). VMAT1 is critical for the transport of monoamine neurotransmitters into secretory vesicles and is indispensable for efficient catecholamine release in response to stimuli (80). Consequently, reduced levels of these proteins would be expected to impair vesicle fusion capability and neurotransmitter loading, thereby offering a mechanistic explanation for the compromised regulated secretion observed after AP-3 depletion. VMAT1 contains a C-terminal dileucine motif (4) that is predicted to interact with the σ1 subunit of AP-3 complex through AlphaFold modelling. While PLA suggested a proximity between VMAT1 and the AP-3 complex (δ subunit), co-immunoprecipitation in PC12 cells further validated this interaction between the AP-3 complex (μ subunit) and VMAT1, supporting the requirement of AP-3 for the loading of VMAT1 into the DCV and for regulated exocytosis of catecholamines.

In addition to these known vesicle components, our proteomic analysis identified additional candidate proteins that may rely on AP-3 for proper trafficking into DCVs. DLK1 serves as a previously unrecognized cargo of the regulated secretory pathway. DLK1 has primarily been studied as a modulator of developmental signaling and cellular differentiation (81). Its presence in VEFs and its secretion in response to stimuli suggest that it may represent a new DCV cargo. While the molecular basis of the interaction between DLK1 and the AP-3 complex is still unclear due to the poorly resolved structure of DLK1, AlphaFold modelling predicts weak interactions of the σ subunit of AP-3 with DLK1, while *in vivo* PLA in PC12 cells indicated proximity between DLK1 and the AP-3 complex (δ subunit).

**Figure 8:**
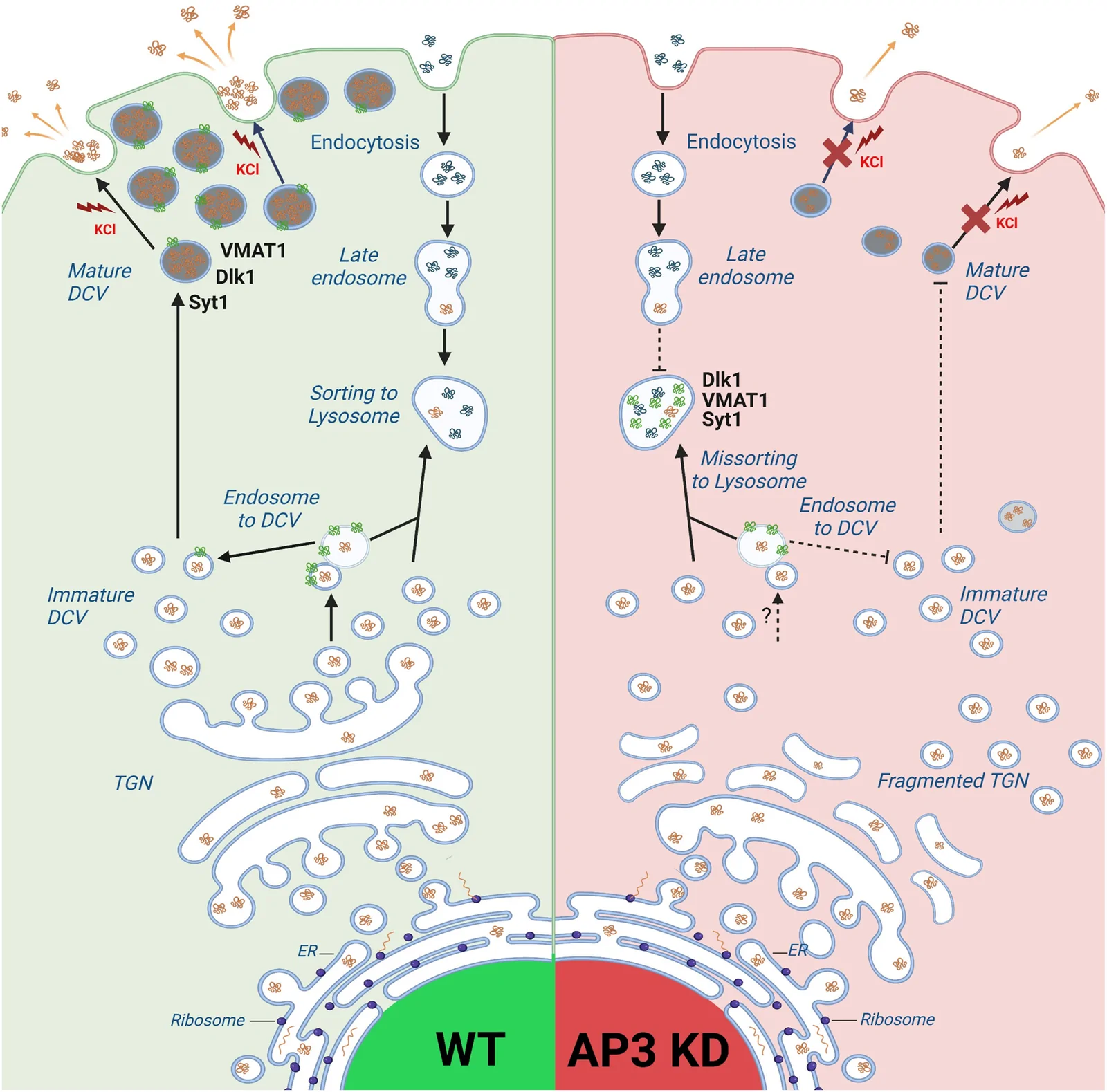
**Plausible mechanisms of AP-3 affecting DCV function.** The presence of AP-3 at both the TGN and endosomes suggests two plausible mechanisms of cargo sorting. At the TGN, AP-3, along with Clathrin, helps form specific sorting sites where DCV cargo is loaded into immature DCVs. These immature DCVs then undergo homotypic fusion and mature, moving farther away from the TGN and getting docked at the plasma membrane for stimulus-coupled release. Alternatively, AP-3 present at endosomes may be responsible for loading DCV cargo into immature vesicles, thereby making the DCVs more condensed and promoting maturation. AP-3 is also responsible for the sorting of SLVs at the endosomes, the recycling of exocytotic vesicles, and clearance *via* the Endolysosomal pathway. This figure shows the vesicle trafficking in WT PC12 cells compared to AP-3 KD cells. The absence of AP-3 inhibits DCV maturation and may lead to stunted transport of specific membrane proteins and Cargo to DCVs, resulting in mis-sorting of membrane proteins to lysosomes. Thus, it affects the regulated exocytosis of DCV at multiple checkpoints.

Collectively, these findings support a model in which AP-3 coordinates multiple trafficking steps required for the formation and functional maturation of DCVs (Fig. 8). In this model, AP-3 contributes to cargo sorting at the trans-Golgi network and participates in trafficking pathways that maintain the appropriate localization of AP-3-dependent preferential DCV membrane proteins. Through these functions, AP-3 facilitates the efficient export of DCV cargo and ensures the incorporation of key membrane proteins, such as SYT-1 and VMAT1, into DCVs. In parallel, AP-3 supports retrograde trafficking pathways that recycle SNARE proteins such as STX-6 to the TGN, thereby facilitating homotypic fusion of immature DCVs and vesicle maturation. Disruption of AP-3, therefore, results in delayed cargo trafficking, altered vesicle composition, and impaired maturation of DCVs. Vesicles arriving at the cell periphery under these conditions exhibit reduced fusion competence, leading to reduced stimulus-dependent secretion. This model highlights AP-3 as an adaptor that not only selects cargo but also coordinates trafficking pathways required for the structural and functional integrity of DCVs.

While the findings suggest that impaired STX6 trafficking contributes to defective DCV maturation, the molecular mechanism linking AP-3 to STX6 recycling remains unclear. It will be necessary to determine whether STX6 represents a direct cargo of AP-3-mediated transport or whether the observed trafficking defect arises indirectly through disruption of upstream sorting pathways. This study also reports previously unidentified AP-3-dependent DCV cargo that requires further characterization to understand their role in the regulated exocytosis of DCVs.

## Experimental procedures

### Cell culture

PC12 cells (from ATCC) were maintained in DMEM media (Gibco, 12100046) supplemented with 10% Horse Serum (Gibco, 16050114), 5% Fetal Bovine Serum (Gibco, F7524) and Antibiotic/Antimycotic (Gibco, 15240062) in a humidified cell culture chamber at 37 °C with 5% CO_2_. The cells were routinely treated with *Mycoplasma* removal agent (MP Biomedical, 30-50044) and used in experiments for no more than nine passages. The SMARTvector rat lentiviral system (Dharmacon) was used to generate inducible shRNA-expressing cells targeting AP-3δ knockdown and coexpressing GFP. The following constructs were used in the study: AP-3sh1, 5′ – TGGTCAAGGGCAGTATCGA – 3′; AP-3sh2, 5′ – GCTGAGAATCCGTACATTA – 3′; AP-3sh3, 5′ – GAAGGTACTGACGTCATCA – 3′.The generation of PC12 cells stably expressing the inducible shRNA constructs, for future reference, AP-3sh1, APsh2, and AP-3sh3, was performed as described previously (22). Briefly, lentiviral transduction containing the virions encoding three different constructs, as mentioned previously, was carried out in PC12 cells in poly L lysine (Sigma-Aldrich, P2636) coated six well plates according to the manufacturer’s protocol (Dharmacon). The cells containing the integrated lentiviral constructs were selected in 2 μg/ml puromycin (Sigma, 118M4079V), constantly replacing the medium for 3 days until resistant colonies were seen. The cells were induced with 5 μg/ml doxycycline (Himedia, 0000-348412) overnight, and highly expressing GFP-positive cells were obtained by fluorescence-associated cell sorting (FACS). The cells were maintained in a medium containing 0.2 μg/ml puromycin without doxycycline.

For experiments, AP-3sh1, AP-3sh2, and AP-3sh3 cells were split and maintained in PC12 media containing 0.2 μg/ml puromycin and 5 μg/ml doxycycline for 5 days to induce the knockdown of AP-3, labelled as +DOX or KD. Simultaneous maintenance of non-induced cells, labeled as mock treated (−DOX), was used as a control for each experiment.

### Mammalian expression constructs

All the expression plasmids used in this study are mentioned in Table S2.

### Transfections, immunofluorescence, and confocal imaging

AP-3sh1 and AP-3sh2 were transfected using METAFECTENE PRO (Biointex, Lot. No. RKP205/RK081621) through the protocol provided by the manufacturer (82). In summary, plasmid DNA and METAFECTENE PRO were mixed separately in DMEM medium without any supplements, mixed and incubated for 20 min at room temperature for complex formation. The complex was added directly to the cells grown in DMEM with 10% horse serum and 5% fetal bovine serum within 4 h of cell seeding. For stable transfection of cells, they were seeded in a 12-well plate and transfected according to the protocol described. After 12 h, the cells were incubated in PC12 media for 24 h. The transfected cells were then transferred to a 60 mm dish and cultured in PC12 media containing 2 mg/ml G418 (GoldBio, G-418-1) for 14 days to select cells stably expressing NPY-mApple and NPY-pHTomato.

For transient transfections to see the intracellular localization, mock treated (−DOX) and AP-3 depleted (+DOX) cells were seeded on a 15 mm coverslip (Assistent/Glaswarenfabrik). As mentioned previously, a complex containing the plasmid and METAFECTENE PRO was added directly to each coverslip within 4 h of seeding. The cells were kept in PC12 media for 12 h post-seeding, then for up to 72 h, after which the cells were used for Immunofluorescence imaging. The transfections carried out were of the plasmids, SYT1 mCherry, Syt4 mCherry, Syt7 mCherry, VMAT1 mCherry, VMAT2 mCherry, Phogrin mCherry, Lyso20 mCherry.

For Immunofluorescence, a previously standardized protocol was followed (83). In brief, mock treated (−DOX) and AP-3-depleted (+DOX) cells were seeded onto PLL coated 12 mm coverslips. Cells were rinsed with 1X PBS (Gibco, 21600-069) and fixed with 4% paraformaldehyde (PFA) (Fisher Scientific, 30525-89-4) for 20 min at room temperature. Post-fixation, cells were again gently rinsed with 1X PBS (3 times for 5 min each) and then permeabilized using 0.1% Triton X-100 (Sigma-Aldrich, 9002-93-1) for 10 min. The permeabilized cells were again rinsed with 1X PBS (2 times for 5 min each) and blocked using 1% bovine serum albumin (BSA) (Sigma, A4503) for 1 h. Following that, the cells were subjected to incubation with a primary antibody overnight. Post incubation with primary antibody, cells were rinsed again with 1X PBS (3 times for 5 min each) and incubated in secondary antibodies(Invitrogen Rabbit Alexa Fluor 647(A-31573), Mouse Alexa Fluor 647(A-31571), Rabbit Alexa Fluor 594(A-11012), Mouse Alexa Fluor 594(A-11005)), with their cognate primary antibodies, followed by gently washed with 1X PBS (3 times for 5 min each). The coverslips were then mounted using Flouroshield (Sigma, F6057) to reduce photobleaching. All images were captured using a Nikon A1HD25 confocal microscope under a 60X oil objective (NA 1.4) at a 2048∗2048-pixel resolution unless mentioned differently.

### Immunoblotting

Mock treated (−DOX) and AP-3 depleted (+DOX) cells were lysed using RIPA buffer (10 mM Tris-HCl (Sisco Research Laboratory Pvt. Limited, 37969/77-86-1), 150 mM NaCl (MP Biomedicals, 194848), 1 mM NaVO_4_ (Sigma-Aldrich, S-6508), 30 mM Na_4_P_2_O_7_ (Sigma-Aldrich, 011K0307), 50 mM NaF (Sigma-Aldrich, 7681-49-4), 1% Nonidet P-40 (Sigma-Aldrich), 0.1% SDS (Affymetrix USB Products, 151-21-3), 1 mM PMSF (Sigma-Aldrich, 52K0052), 1% Triton X-100 (Sigma-Aldrich), 0.5% C_24_H_39_NaO_4_ (Sigma-Aldrich, 145224-92-6), and dissolved protease inhibitor cocktail (Roche Diagnostics, 11714900) in water, pH 7.4). The protein contents of the cell lysate were quantified using a BCA Protein Assay Kit (TAKARA) following the manufacturer’s protocol. The samples were separated using a 10% SDS-polyacrylamide gel and transferred onto nitrocellulose membranes. After blocking with 5% BSA (Sigma-Aldrich), membranes were incubated with primary antibodies, followed by incubation with secondary antibodies (Peroxidase AffiniPure Goat Anti-Rabbit IgG (H + L) Jackson Immuno Research 111-035-003, Peroxidase AffiniPure Goat Anti-Mouse IgG (H + L) Jackson Immuno Research 115-035-003). The membrane was washed thrice for 10 min after primary and secondary antibody incubation to remove excess antibodies. ECL was prepared using the previously described protocol, and bands were visualized using the Uvitec Mini HD9 gel imaging system (84). Densitometry quantifications were done using ImageLab (6.0.1) (85).

### Stimulus coupled secretion assay

A stimulus-coupled secretion assay was performed in PC12 cells according to a previous protocol (22, 36). In brief, mock treated (−DOX) and AP-3 depleted (+DOX) cells were plated in a 60 mm dish (Tarson) at a confluency of 90%. 24 h post cell seeding, mock treated (−DOX) and AP-3 depleted (+DOX) cells were incubated with basal buffer (150 mM NaCl, 5 mM KCl, 2 mM CaCl_2_, 10 mM HEPES; pH 7.4) and stimulation buffer (150 mM NaCl, 100 mM KCl, 5 mM BaCl_2_, 10 mM HEPES; pH 7.4) for 20 min at 37 °C in a humidified cell culture chamber with 5% CO_2_. The supernatant was then collected, whereas cell lysates were prepared, as mentioned previously, for Immunoblotting. Supernatants and cell lysates were collected and run in SDS-PAGE and probed with CgB (Proteintech, 14968-1-AP,1:5000), DLK1(Proteintech,10636-1-AP), GAPDH(Proteintech,60004-1-Ig,1:10000), and B-Tub (Proteintech,10094-1-AP,1:20000) for AP-3sh1, AP-3sh2, and AP-3sh3.

A stimulus-coupled secretion assay was also performed using a different secretagogue to assess regulated secretion using KCl (36). Stably selected cells of NPY-mApple in an AP-3sh1 background were also subjected to a stimulus-coupled secretion assay (83). In summary, mock treated (−DOX) and AP-3 depleted (+DOX) cells harboring NPY-mApple were seeded in a 6-well plate at 90% confluency and incubated with basal buffer (150 mM NaCl, 5 mM KCl, 2 mM CaCl_2_, 10 mM HEPES; pH 7.4) and stimulation buffer (55 mM NaCl, 100 mM KCl, 2 mM CaCl_2_, 10 mM HEPES; pH 7.4) for 20 min at 37 °C in a humidified cell culture chamber with 5% CO_2_. Collected supernatants and cell lysates were processed as mentioned previously. For the plate reader assay, 10 μl of the cell lysate and the supernatants were diluted 1:10 in 1X PBS (Gibco). 100 μl of the solution was loaded in a dark 96-well plate (Tarson) in duplicates, along with subsequent controls for background subtraction. NPY-mApple fluorescent signals were then recorded using the TECAN plate reader (Ex λ-568 nm, Em λ-592 nm), subtracting them from the autofluorescence of the plate itself and the percentage of secretion was analyzed (86, 87).

### Constitutive secretion

To check the constitutive release of proteins in basal conditions, secreted fractions from unstimulated cells were analyzed by SimplyBlueTM SafeStain (Invitrogen, LC6060) Coomassie staining (35, 36). In brief, mock-treated (−DOX) and AP-3-depleted (+DOX) cells were seeded in 60 mm culture dishes at a density of 10^6^ per dish. The cells were incubated with basal buffer (150 mM NaCl, 5 mM KCl, 2 mM CaCl_2_, 10 mM HEPES; pH 7.4) for 3 h, during which the supernatants and cell lysates were collected and processed as mentioned previously in the report. The samples were resolved using a 10% SDS-polyacrylamide gel. The gel was washed with Milli-Q water twice and then stained with SimplyBlueTM SafeStain (Invitrogen) for 1 h at room temperature with gentle shaking. To remove the background, the gel was washed with Milli-Q water twice and later imaged in the Azure c300 gel imaging system, and all the automatically detected bands were quantified using ImageLab (6.0.1). Furthermore, for estimating constitutive secretion using a marker, Immunoblotting was carried out and probed for HSP90 (Proteintech,13171-1-AP,1:1000). Bands were visualized using the Uvitec Mini HD9 gel imaging system (84).

### Transmission electron microscopy

Sample preparation for transmission electron microscopy was carried out according to a previous protocol (83). Briefly, mock treated (−DOX) and AP-3 depleted (+DOX) cells from AP-3sh1 and AP-3sh2 were seeded in a 100 mm dish. The cells were fixed overnight with routine fixative, 2% PFA (Fisher Scientific) and 2.5% glutaraldehyde (Sigma – Aldrich, MKBG0637V) in 0.15 M sodium cacodylate buffer (Sigma – Aldrich, C0250). Cells were then pelleted at 10,000 rpm for 10 min in fresh fixative. Post-fixation was done for 1 h in 1% osmium tetroxide (Sigma, 05500), followed by three washes with 0.15 M sodium cacodylate buffer. Subsequent dehydration was done through graded ethanol incubations (10 min each of 50%, 70%, 90%, 2 × 100%). Pelleted cells were then incubated in propylene oxide (Tokyo Chemical Industry, E0016) (2 × 20 min), followed by overnight infiltration in a 1:1 mixture of propylene oxide and Araldite 502 (Ted Pella, 18060). The samples were subsequently embedded in a mixture of Araldite 502, DDSA (Ted Pella, 18022), and DMP-30 (Ted Pella, 18042) as per a previous protocol (88) and polymerized at 60 °C for 72 h. Ultrathin sections were cut on a Leica UCT ultramicrotome, picked onto carbon-coated copper grids (Electron Microscopy Sciences), stained with 10% Uranyl acetate (Sisco Research Laboratory, 81405), and counter-stained with Sato’s lead citrate (Electron Microscopy Sciences, 17800) and imaged using JEOL JEM1400-plus transmission electron microscope. The images were used for morphometric analysis in ImageJ software. For morphometric analysis, two perpendicular line segments were drawn inside the dense core and across the whole DCV, and the average of these was taken as the diameter. To calculate the DC/DCV area, the area of the DC and DCV was calculated from the diameter, and a ratio of these represented the DC area component of the DCV. Similarly, the Halo area/DCV area was calculated by subtracting the DC area from the total DCV area and represented as a fraction of the DCV area. Furthermore, intracellular DCV distribution was obtained by measuring the shortest distance of the DCV from the cell membrane. Also, the individual DCVs were also manually counted, and their distribution as per the distance from the plasma membrane was also analyzed and represented.

### Retention using selective hook assay and analysis

1.5 million Cells were seeded in each of two 60-mm dishes with seven coverslips. After 4 h, cells were transfected with NPY-SBP-MCherry (Cargo) and KDEL-Str (Hook) plasmids (a kind gift from CS Asensio lab) in a 1:3 ratio (42). After 8 h, the transfection media were replaced with full media, and the cells were grown for 48 h before the experiment. 50 mM Biotin(Sigma, B4501-1G) to the final concentration in Opti-Mem was used to replace the full DMEM for the experiment, and coverslips from each condition were fixed at five different time points (0, 20, 40, 60, and 90 min) post Biotin addition (89). Then, fixed coverslips were subjected to Immunofluorescence staining for Golgin 97(Proteintech,12640-1-AP, 1:1000) and visualized using a confocal microscope as described previously. For the analysis, Mander’s coefficient tM1 was used to measure the colocalization between Golgin 97(Golga1) and Cargo (tagged with mCherry) for all the time points. To measure the Rate of entry and the Rate of exit of Cargo at Golgi, the slope was calculated as,

Rateofentry=(tM1at20min)−(tM1at0min)/20−0

RateofExit=(tM1at40min)−(tM1at20min)/40−20

Where tM1 corresponds to the colocalization Manders ’ coefficient between Golgi and NPY mCherry. These entry and exit rates were further normalized to the average rate of entry and exit in mock cells, and the absolute value for the rate of exit was represented.

### Brefeldin A assay

In a 12-well plate with 15 mm coverslips, 200,000 cells were seeded using the mock treated (−DOX) and AP-3 depleted (+DOX) cells. After 24 h, the cells were treated with 2.5ug/ml of BFA in DMEM complete media (10%HS+5%FBS) for 15 min at 37 °C inside a CO_2_ incubator. The first coverslip was washed with 1X PBS and fixed with 4% PFA before the treatment with BFA (Sigma, B6542) to serve as the control for the experiment. After the BFA treatment, cells were washed with 1X PBS twice, and the second coverslip was fixed with 4% PFA, while the other coverslips were incubated with DMEM complete media without any BFA (this is called the washout step). Further, the coverslips were fixed at 20 min and 40 min post-washout, respectively. Once all the coverslips were fixed, immunofluorescence was done using a Golgin 97 antibody to visualize the Trans Golgi network using a confocal microscope. This protocol was adapted from (90). For analysis, the puncta visible in the images were counted using the analyze particle plugin of image J and were normalized per cell (91). Multiple t-tests were performed for each condtiton.

### SILAC, vesicle-enriched fraction preparation, Immunoblotting, and mass spectrometric analysis

For Stable Isotope Labelling by Amino Acids in Cell Culture (SILAC) experiments, PC12 cells were cultured for at least 3 weeks to achieve metabolic labelling in “SILAC medium”. The “SILAC medium” consisted of DMEM (Silantes) supplemented with 5% (vol/vol) dialyzed Fetal Bovine Serum (Silantes) and 10% (vol/vol) Horse Serum (Silantes) and either “heavy” amino acid (l-arginine - ^13^C_6_^15^N_4_ (50 mg/l) and l-lysine - ^13^C_6_^15^N_2_ (100 mg/l)) (Silantes) or “light” amino acid (l-arginine - ^12^C_6_^14^N_4_ and l-lysine - ^12^C_6_^14^N_2_) (Sigma-Aldrich). The average incorporation efficiency was calculated to be ∼98%, as seen by mass spectrometry.

For vesicle-enriched fraction preparation, the protocol was followed as described previously (22, 48). To summarize, two fully confluent 500 cm^2^ dishes (Corning) were seeded with mock treated (−DOX) and AP-3 depleted (+DOX) cells for each preparation. At 4 °C, each dish was rinsed with 1X PBS and scraped with 12 ml of Buffer A (0.1 M MES, pH 6.5 [adjusted with NaOH], 0.2 mM EGTA, and 0.5 mM MgCl_2_). The cells were then homogenized using a motorized Dounce homogenizer (25 strokes) and passed through a syringe using a 21G X 2” needle. 200 μl of the homogenized solution was kept separately and processed for cell lysate, whereas the rest of the solution was centrifuged at 4500 rpm for 30 min. The supernatant was treated with 50 μg/ml ribonuclease A (MP Biomedical) for 60 min at 4 °C. The digested ribosomes were pelleted by centrifugation at 4500 rpm for 15 min. The resulting supernatant was centrifuged at 55,000 rpm for 45 min (MLA 80 rotor, Beckman Coulter). The pellet was resuspended in 350 μl of Buffer A and again subjected to homogenization using a 1 ml Dounce homogenizer (69 strokes) and mixed with an equal volume of FS buffer (12.5% [wt/vol] Ficoll, 12.5% [wt/vol] sucrose in Buffer A). The solution was then pelleted, and the supernatant was diluted 5 times in Buffer A and centrifuged at 35,000 rpm for 35 min (TLA-110 rotor, Beckman Coulter). The pellet was then identified as the vesicle-enriched fraction (VEF) and processed accordingly for mass spectrometry and Immunoblotting.

For Immunoblotting, AP-3sh1 and AP-3sh2 cells were used. In brief, both mock treated (−DOX) and AP-3 depleted (+DOX) cells were seeded in 2 × 500 cm^2^ dishes and subjected to vesicle-enriched fraction preparation, as mentioned above. 2X non-reducing SDS buffer (4% [wt/vol] SDS and 10 mM Tris-HCl, pH 8.0) (92) was mixed in a 1:1 ratio with the cell lysate, and the subsequent solution was passed through the QIA shredder (Qiagen). For the VEF, 50 μl 1X non-reducing SDS buffer was used for resuspending the pellet. The samples were heated at 95 °C for 5 min and processed, as mentioned before, for Immunoblotting. In all cases, 20 μg of cell lysate was loaded, whereas 2.5 μg of VEF was loaded. The blots were processed accordingly and probed for targeted proteins Clathrin Heavy chain (CHC, Proteintech 26523-1-AP,1:2000), SYT1(Synaptic systems 105011,1:2500), ZnT3(Proteintech 17363-1-AP, 1:2500), VMAT1(Proteintech 20340-1-AP, 1:2000), AP-3m1(Proteintech 12114-1-AP, 1:2000), DLK1(Santa Cruz SC-25437,1:500), EF2 (loading control) (Santa Cruz sc-166415, 1:2000). Analysis for the blots was performed in imageJ normalizing with EF2 as loading control. For mass spectrometry, metabolically “heavy” and “light” cells were cultured accordingly. For the first experiment in AP-3sh1, mock treated (−DOX) cells were cultured in a “heavy” medium, whereas AP-3 depleted (+DOX) cells were cultured in a “light” medium. In the second experiment, mock treated (−DOX) cells were cultured in a “light” medium, whereas AP-3 depleted (+DOX) cells were cultured in a “heavy” medium. The VEFs from each condition were resuspended in 30 μl of 1X non-reducing SDS buffer and then mixed. The samples were run in a NUPAGE precast 12% gel (Invitrogen) and stained with SimpleSafe Blue Stain (Thermo Fisher). Each lane in the gel was cut into eight pieces and then analyzed for mass spectrometry. The resolved proteins were reduced, alkylated, and digested in-gel with trypsin, and then the resulting peptides were concentrated and desalted using Stage tips (93). Tryptic peptides were analyzed by LC-MS/MS using a Q-Exactive coupled to an RSLCnano3000 UHPLC (Thermo Fisher). Peptides were resolved using a 50-cm EASY-Spray PepMap C18 column (Thermo Fisher) with data acquired in a data-dependent acquisition manner.

Data was processed using MaxQuant v2.1.4.0 using Andromeda to search a Uniprot *Rattus norvegicus* database (downloaded 7 April 2014, 27,344 entries). N-terminal protein acetylation and methionine oxidation were set as variable modifications, carbamidomethyl cysteine was set as a fixed modification, and both LFQ and iBAQ were enabled. Oxidised methionine was set as a variable modification and carbamidomethyl cysteine as a fixed. Default false discovery rate calculations were used as provided by MaxQuant. The raw data files were processed using MaxQuant (with requantify and match between runs features enabled). The primary outputs for each SILAC comparison were a list of identified proteins, a relative abundance ratio (heavy/light), and the number of quantification events (count). Each MaxQuant output file was formatted identically: reverse hits, proteins identified only by site, common contaminants, and proteins with no gene name were removed. Where indicated, ratios were normalised on protein average. The relative abundance for enriched protein was calculated using the iBAQ method as implemented in MaxQuant. The median iBAQ value was calculated for each protein and then normalized to the iBAQ of clathrin heavy chain (one of the most abundant protein in the VEF preparation, assigned an arbitrary “abundance score” of 1,000,000). For each protein the average ratio was subtracted by median of average and divided by the robust standard deviation (1.4826∗ median absolute deviation from the median) for Z-score calculations. The *p*-values were calculated based on the Z-scores and the list of protein hits is provided as Table S1 for reference. The analysis is based on the works previously published (94, 95).

### Dextran uptake assay

A previously published protocol was used to examine exocytosis modes (60). In brief, mock treated (−DOX) and AP-3 depleted (+DOX) cells were washed once with 1X PBS and twice with basal buffer (150 mM NaCl, 5 mM KCl, 2 mM CaCl_2_ and 100 mM HEPES, pH 7.4) and then incubated for 5 min with 50 μM dextran (10 and 40 kDa, Invitrogen D1817 and D1842) in 100 mM KCl containing stimulation buffer (55 mM NaCl, 100 mM KCl, 2 mM CaCl_2_ and 100 mM HEPES, pH 7.4) at 37 °C. The unbound dye was washed out with the previously mentioned bath solution (without Ca^2+^) and immediately fixed with 4% PFA. Confocal images were captured, and, for analysis, the total number of dextran fluorescent puncta per cell was determined by thresholding and using the Analyse Particles plugin in ImageJ.

### Catecholamine uptake assay

Mock-treated (−DOX) and AP-3-depleted (+DOX) cells, cultured in six-well plates coated with poly-l-lysine and reaching 80% confluence, were exposed to 1 μCi [3H] l-norepinephrine for a 3-h incubation period. Subsequently, they underwent two washes with release buffer and were incubated for 15 min at 37 °C in either release buffer or stimulation buffer. Subsequently, supernatants were collected, and the cells were lysed in lysis buffer. Both the supernatants and lysates were combined with a liquid scintillation cocktail, and the radioactivity was measured using a Beckman liquid scintillation counter. The experiments in Figures 7 and S5 represent biological replicates conducted independently thrice. This protocol was adapted from (22).

### Zinquin ethyl ester assay

To visualize zinc uptake, we used a previously published protocol (60) with the Zinquin ethyl ester (Abcam, GR241459-21) dye. Mock-treated (−DOX) and AP-3-depleted cells (+DOX) were seeded onto 15 mm coverslips in 12-well plates and incubated overnight. The next day, cells were incubated with a solution of ZnSO_4_(SRL, 7446-20-0) containing 50 μM ZnSO_4_, 10 mM Sodium pyruvate (Gibco, 11360-070), and 10 mM glucose (MP Biomedical, 194672) in HBSS (with no calcium and magnesium) for 1 h at 37 °C. Later, the cells were washed with 1X PBS and incubated with 50 μM Zinquin ethyl ester (in 10 mM glucose in PBS) for 20 min at 37 °C. After which, the cells were washed multiple times (at least twice) with 1X PBS and fixed with 4% PFA for 10 min. These coverslips were imaged in the Nikon Eclipse Ti2 at 100X oil (NA 1.30).

### Endolysosomal assay

In this assay, 200,000 cells were seeded on coverslips in a 12-well plate from both mock-treated (−DOX) and AP-3-depleted (+DOX) cells and incubated at 37 °C with 1% 10 kDa Dextran (50 μM) in DMEM incomplete media (*i.e.*, no serum or antibiotics) for 30 min. After the cells were washed twice with 1X PBS, the individual coverslips were fixed at 0-, 30-, and 60-min post-washing, respectively, with 4% PFA. Confocal images were captured, and, for analysis, the total number of dextran fluorescent puncta per cell was determined by thresholding and using the Analyse Particles plugin in ImageJ.

### Protein-protein interaction using ColabFold

Protein sequences for the AP-3 complex subunits SYT1, DLK1, and VMAT1 specific to rats were obtained from UniProt. To run the modelling, the protein sequences were fed into ColabFold (62) without changing any default settings. After modelling was completed with ColabFold using the different algorithms, the generated predictions were ranked 1 to 5. Based on parameters such as pLDDT, pTM, ipTM, and PAE score, the most suitable interaction was selected. The PDB files generated by these algorithms were analyzed in ChimeraX and PyMOL to identify interacting amino acids. The interactions reported were thresholded at a bond length of 4 Å or less, including both polar and non-polar contacts (96).

### Colocalization and statistical analysis

All images captured by Nikon A1HD25 confocal microscope under a 60X oil objective (NA 1.4) at a 2048∗2048-pixel resolution were deconvoluted at the time of capture. Image J plugin JaCoP (Just another Colocalization plugin) was used for colocalization. The tM1 values from the colocalization were then converted to percentages by multiplying the decimal values by 100 and reported as percentage colocalization. Object-based colocalization analysis was also performed using JaCoP, selecting object-based methods that calculated colocalization based on the center-of-mass particle coincidence. These values were converted to percentages for representation. All quantitative analyses were performed in Prism 8.0. Graphs were presented as mean ± SD and statistically compared using a two-tailed unpaired *t* test (GraphPad Prism 8.0) when the data were normally distributed. For cases where the data failed the normality test, statistical comparisons between the two groups were performed using the two-tailed Mann-Whitney *U* test (GraphPad Prism 8.0). Differences between groups were considered statistically significant for *p* values < 0.05. All the experimental data shown were analyzed from at least three independent experiments or at least eight cells.

### Proximity ligation assay

The PLA (69) was performed using the Duolink In Situ Red Starter Kit (Mouse/Rabbit DUO92101; Sigma-Aldrich) to assess *in vivo* interactions among AP-3δ and SYT1, AP-3δ and VMAT1-GFP, and AP-3δ and DLK1 in WT PC12 cells. The cells were fixed in 4% PFA for 20 min and washed twice with PBS. 0.2% TritonX-100 in PBS was used to permeabilise cells. The primary antibodies used were AP-3δ (AB_2056641, Dilution 1:500), DLK1 (10636-1-AP, Dilution 1:500), Synaptotagmin 1 (105011, Dilution 1:1000), and GFP (50430-2-AP, Dilution 1:500). The protocol was followed as described (69). All images were captured using a Nikon A1HD25 confocal microscope with a 60X oil objective (NA 1.4) at a 1024∗1024-pixel resolution as Z-stack images with a step size of 0.3 μm. For representation, the images were Z-projected at maximum intensity. The PLA signal corresponds specifically to the red dots in the images. For the negative control, we used a no-antibody control, which showed no PLA signal.

### Native Co-immunoprecipitation

We have used the soft elution method for native co-immunoprecipitation experiments as described (93). The beads were washed four times with PBS-T (phosphate-buffered saline containing 1% Triton X-100), followed by a single wash with PBS to remove detergent. Bound proteins were eluted by incubating the beads in 100 μl of soft elution buffer (0.2% SDS, 0.1% Tween-20, 50 mM Tris-HCl, pH 8.0) at 25 °C for 7 min with shaking. The elution was repeated once, after which the eluates were combined and centrifuged at 16,000×*g* for 1 min to remove residual beads. IP eluates and IgG IP samples were loaded on 10% SDS-PAGE gel and analyzed by immunoblot. 1% of the initial homogenates was loaded as input.

## Data availability

All data that support the findings of this study are included within the article and its supporting information. The raw data supporting the conclusions will be made available from the corresponding author upon request.

## Supporting information

This article contains supporting information.

## Conflict of interest

The authors declare that they have no conflicts of interest with the contents of this article.
