# Supplementary material for: AP-3 complex sorts preferential cargo to govern dense core vesicle function in neuroendocrine cells
Source: J Biol Chem. 2026 Aug 5;302(9):113396. doi: 10.1016/j.jbc.2026.113396 (PMC13546849; doi:10.1016/j.jbc.2026.113396)
Supplement: Supplementary file 1 [file mmc1.docx]

**AP-3 complex sorts preferential cargo to govern dense core vesicle function in neuroendocrine cells**

Shashank Saxena^1^, Chandramouli Mukherjee^1^, Vinayak Ghosh^1^, Badal Singh Chauhan^2^, Bhavani Shankar Sahu^1*^

1. National Brain Research Centre, Manesar, Gurgaon, Haryana 122052, India

2. Department of Biological Sciences, Tata Institute of Fundamental Research, Mumbai, Maharashtra 400005, India

* Bhavani Shankar Sahu

**Email:** bssahu@nbrc.ac.in

**Supporting Information**

**List of material Included:**

**Figures S1-S8**

**Table S1. Mass Spectrometry raw data.**

**Table S2. Expression plasmids used in the study.**

**Figure S1. Stimulus-coupled secretion experiments reveal compromised DCV exocytosis in AP-3-depleted cells while constitutive secretion remained unaffected.** (A) Representative immunoblot showing AP-3 knockdown in AP-3 shRNA3 cells. (B) Representative immunoblot for the secretion of CgB in mock treated (-DOX) and AP-3 depleted cells (shRNA2) (+DOX) using BaCl_2_ as a secretagogue indicating a similar defect in the BaCl_2_ induced release of CgB. (N=3) (C) Representative immunoblot for the secretion of CgB in mock treated (-DOX) and AP-3 depleted cells (shRNA3) (+DOX) using BaCl_2_ as a secretagogue indicating a similar defect in the BaCl_2_ induced release of CgB. (N=3) (D-E) Immunoblot showing no change in the constitutive release of HSP90 (protein unique to constitutive secretory pathway) in mock treated (-DOX) and shRNA1 and shRNA2 AP-3 depleted cells (+DOX) (N=2). (F-G) Representative gel picture shows the global constitutive signature and the corresponding quantification considering the auto-detected band intensities in Image-lab in shRNA1 and shRNA2 AP-3 depleted cells where each dot represents individual band intensities indicating no change in the constitutive signature. (N=3, two-tailed Mann–Whitney U test). Data is shown as mean ± SD. (H) Representative immunoblot shows no change in the case of CgB secretion for mock treated (-DOX) and doxycycline treated scrambled shRNA cells (+DOX) (N=2). Data are shown as mean ± SD; ns, non-significant.

**Figure S2. AP-3 affects the maturation of DCVs in PC12 cells.** (A-B) Graphs for the relative frequency distribution of the DCVs with respect to DC diameter and DCV Diameter from the transmission electron microscopy images for mock treated (green, -DOX (332 DCVs)) and shRNA1 AP-3 depleted cells (Red, +DOX (403 DCVs)). (C-D) Representative transmission electron microscope images showing for mock treated (-DOX) and shRNA2 AP-3 depleted cells (+DOX). Scale bar: 500nm, inset: 100nm. (E) Graphs showing an increase in DC diameter (-DOX (70 DCVs with mean DC diameter of 63.77 ± 9.6nm) and +DOX (115 DCVs with mean DC diameter of 69.74 ± 15.73nm) (two-tailed Mann–Whitney U test, **, *p*=0.0029). (F) graph showing an unaffected DCV diameter (-DOX (72 DCVs with mean DC diameter of 99.55 ± 27.11nm) and +DOX (67 DCVs with mean DC diameter of 104.9 ± 26.18nm, two-tailed Mann–Whitney U test, ns, non-significant). (G) This Graph depicts analysis of the DC area/ DCV area with a significant increase in the DC area in AP-3 depleted cells. (For -DOX (72 DCVs) the DC/DCV area mean was 0.3843 ± 0.1054, for +DOX (76 DCVs) the DC/DCV area mean was 0.4268 ± 0.07383; two-tailed unpaired Student’s t-test, **, *p*=0.005) (H) Quantification for the halo area/ DCV area (where the halo refers to the clear volume between the membrane and core (DCV minus DC area)) shows a significant decrease in the halo area in AP-3 depleted cells (For -DOX (72 DCVs) the Halo/DCV area mean was 0.6157 ± 0.1054 and for +DOX (76 DCVs) the Halo/DCV area mean was 0.5723 ± 0.07383, two-tailed unpaired test, **, *p*=0.005). (I) graph showing DCVs and their distance from plasma membrane increasing farther in case of AP-3 KD (For -DOX (96 DCVs) the mean distance from membrane was 0.4630 ± 0.5419µm, for +DOX (117 DCVs) the mean distance from membrane was 0.9687 ± 0.8128µm, two-tailed Mann–Whitney U test, ****, *p*<0.0001). (J) graph for the number of DCVs showing a trend towards decreased number of DCVs (two-tailed Mann–Whitney U test). (n=9-10 cells for each condition (-DOX (DCV count mean was 25.60 ± 20.75) and +DOX (DCV count mean was 17.75 ± 8.396) assessed by two tailed unpaired Student’s t-test). (K-L) Representative confocal images and quantification showing an increase in colocalization (% colocalization) of STX6 (Red) with CgB (DCV marker) (Green) (N=3, n=10 cells each, two tailed unpaired Student’s t-test, ****p*<0.001). Scale bar: 5µm. Data are shown as mean ± SD.

**Figure S3. AP-3 affects the dynamic organization of the trans-Golgi network and its subsequent sorting of DCV cargoes.** (A-B) Representative confocal microscope fluorescent images for mock treated (-DOX) and shRNA2 AP-3 depleted (+DOX) cells, where Golga1 is green and NPY is red. Scale bar: 10µm. (C) Graphs showing tM1 (Mander’s coefficient 1) of NPY-mCherry and Golga1 for mock treated (Green) and shRNA2 AP-3 depleted cells (Red). (two tailed unpaired Student’s t-test) (D) Bar graph for the Rate of entry of Cargo at the Golgi per minute. (two tailed unpaired Student’s t-test) (E) Bar graph showing a reduction in Cargo's exit rate from the Golgi per minute due to AP-3 KD. (two tailed unpaired Student’s t-test) (N=3 independent experiment, n=8-10 cells each) (F) Representative confocal images for mock treated and shRNA2 AP-3 depleted cells treated with Brefeldin A (dis-assembles Golgi) showing a defect in the Golgi reassembly upon AP-3 Knockdown. Scale bar: 10µm. (Data are shown as mean ± SD, **p*<0.05, ***p*<0.01, ****p*<0.001; ns, non-significant).

**Figure S4. AP-3 plays a selective role in sorting membrane proteins and also affects the trafficking of vesicles post-Golgi in PC12 cells.** (A) WB validates the abundance in the cell homogenate and VEFs for the proteins deficient in MS for mock treated (-DOX) vs shRNA2 AP-3 depleted cells (+DOX). (B) Representative confocal images showing no significant changes in the colocalization (tM1 represented as % colocalization) of DLK1 (red) with the synaptic-like vesicle marker synapsin-2 (green) in mock treated (-DOX) and shRNA-1 AP-3 depleted cells (+DOX). Scale bar, 5 µm (n=10 cells each, two-tailed unpaired t-test). (C) Representative fluorescent images of the mock treated (-DOX) and shRNA2 AP-3 depleted cells (+DOX) showed reduced Zinquin ethyl ester dye uptake. White arrows indicate zinquin dye puncta, Scale bar: 5µm. (D) The graph depicts approximately fivefold greater uptake of nor-epinephrine in the mock treated cells (-DOX) after adding the counts in both the cells and the medium compared to the shRNA2 AP-3 depleted cells. (N=3, two tailed unpaired Student’s t-test, ****, *p*<0.0001). (E) In mock treated (–DOX) cells, release of [^3^H] norepinephrine was strongly dependent on BaCl_2_ stimulation, but in the AP-3-depleted cells (+DOX), the effect of BaCl_2_ was insignificant. (N=3, independent experiment, Data shown as mean ± SD, ****, *p*<0.0001 level assessed by analysis of variance with Tukey’s post-hoc test; n.s. indicates no statistical significance.) (F-G) Representative images and quantification for Dextran 40kDa show impaired full exocytosis as the puncta per cell reduces in shRNA2 AP-3 depleted cells (Red, +DOX) w.r.t mock treated cells (Green, -DOX). (N=3, n=20 cells each, two tailed unpaired Student’s t-test). (H-I) Representative confocal images and quantification for the Endolysosomal experiment using Dextran 10kDa shows a defect in the clearance of the dextran puncta (No. of Puncta/cell) in both the 30 mins and 60 mins in case of shRNA2 AP-3 depleted cells. (N=3, n=20 cells, two tailed unpaired Student’s t-test). Data are shown as mean ± SD, **p*<0.05, ***p*<0.01, ****p*<0.001; ns, non-significant).

**Figure S5. Colocalization of SYT1, DLK1 and VMAT1 (Red) with DCV(Green) reduces in AP-3 knockdown.** (A-F) Representative confocal images and quantification showing colocalization of specific proteins like SYT1, DLK1 and VMAT1 with DCV marker CgB in mock treated (-DOX) vs shRNA2 AP-3 depleted (+DOX) cells indicates a decrease in colocalization upon AP-3 depletion. (n=10 cells from 3 independent experiments for both mock treated (-DOX) and shRNA2 AP-3 depleted cells (+DOX), two tailed unpaired Student’s t-test, Data are shown as mean ± SD, **p*<0.05, ***p*<0.01, ****p*<0.001; ns, non-significant).

**Figure S6. AP-3 preferentially traffics certain membrane proteins to DCVs, while allows other cargoes to be sorted to DCVs.** Representative confocal images for the colocalization of other membrane proteins which are unaffected upon AP-3 depletion. (A-J) NPY-mApple, Phogrin-mCherry, SYT4-mCherry, and SYT7-mCherry (Red) colocalization (tM1 represented as % colocalization with DCV marker CgB (Green) does not change significantly in mock treated (-DOX) and shRNA1 AP-3 depleted cells (+DOX). For NPY-CgB (n=10 cells for mock and n=8 cells for shRNA1 AP-3 depleted cells). For Phogrin-CgB (n=10 cells each), For SYT4-CgB, SYT7-CgB (n=10 cells each, two tailed unpaired Student’s t-test). Data shown as mean ± SD, **p*<0.05, ***p*<0.01, ****p*<0.001; ns, non-significant.

**Figure S7. Prefential cargo gets missorted to the lysosomes upon AP-3 depletion.** (A-B) Colocalization of DCV marker CgB (Green) with Lysosomal marker Lyso20-mCherry (LAMP1) (Red) shows no significant change. (C-H) Colocalization of SYT1, DLK1 and VMAT1-mCherry (Green) with lysosomal marker Lyso20-mcherry/ Lyso20-BFP (for VMAT1) or LAMP1 (Red) increases with AP-3 knockdown, indicating mis-sorting to the lysosomes. (n=10 cells or 3 independent experiments for both mock treated (-DOX) and shRNA2 AP-3 depleted cells (+DOX), two tailed unpaired Student’s t-test, Data are shown as mean ± SD, **p*<0.05, ***p*<0.01, ****p*<0.001; ns, non-significant).

**Figure S8. Protein-protein interactions of AP-3 complex with Specific cargoes.** (A) Colabfold generated protein-protein interactions of AP-3 subunits with Syt1, Dlk1, and VMAT1. The table depicts the interactions and the motifs involved. (B) AP-3 subunit m1 interacts with Syt1 using a tyrosine motif, and the respective figure shows the interaction site and the PAE plot. (C) AP-3 subunit s1 interacts with VMAT1 using a Di-leucine motif, and the respective figure shows the interaction site and the PAE plot. (D) AP-3 subunit s2 interacts with Dlk1 using an acidic-rich cluster motif, and the respective figure shows the interaction site and the PAE plot. (E-H) Representative confocal images of proximity ligation assay in PC12 cells for showing proximity between Syt1(F), VMAT1(G), and Dlk1(H) with AP3δ, where the red dots indicate the interactions and (E) is the negative control (No antibody). White arrows represent the PLA puncta. (I) AP-3mu subunit co-immunoprecipitation blots in wild type PC12 cells showing a pulldown of the AP-3mu subunit and VMAT1. The blots on the right are represented to indicate the bands specific to AP-3mu subunit in the cell lysates of mock treated (-DOX) vs the AP-3 depleted (+DOX) cells.

**Table S1. Mass Spectrometry raw data.**

**Table S2. Expression plasmids used in the study**
