# Supplementary material for: AP-3 complex sorts preferential cargo to govern dense core vesicle function in neuroendocrine cells
Source: J Biol Chem. 2026 Aug 5;302(9):113396. doi: 10.1016/j.jbc.2026.113396 (PMC13546849; doi:10.1016/j.jbc.2026.113396)
Supplement: Supplementary file 2 [file mmc3.docx]

**Table S2. Expression plasmid used in this study**

| NAME | MAKE | Catalog No. |
| --- | --- | --- |
| Synaptophysin-pHTomato | A kind gift from Yulong Li Lab, China | N/A |
| NPY-mApple | Addgene | 83498 |
| mCherry-Lysosomes-20  BFP-lysosome-20 | Addgene  Addgene | 55073  55308 |
| VMAT1-mCherryN1, VMAT1-EGFPN1 | A kind gift from Tom Carter’s Lab, London | N/A |
| VMAT2-mCherryN1 | A kind gift from Gary Miller’s Lab, US | N/A |
| SYT1-mCherryN1 | A kind gift from Ed Chapman’s lab, US | N/A |
| Syt4-mCherryN1 | A kind gift from Ed Chapman’s lab, US | N/A |
| Syt7-mCherryN1 | A kind gift from Ed Chapman’s lab, US | N/A |
| Phogrin-mCherryN1 | A kind gift from John Paul Luzio’s Lab, UK | N/A |
| NPY-mCherry-SBP | A kind gift from Cedric S. Asensio's Lab, US | N/A |
| stim/sp-str-linker-kdel-hook | A kind gift from Cedric S. Asensio’s Lab, US | N/A |
